# Digitally-embroidered liquid metal electronic textiles for wearable wireless systems

**DOI:** 10.1038/s41467-022-29859-4

**Published:** 2022-04-21

**Authors:** Rongzhou Lin, Han-Joon Kim, Sippanat Achavananthadith, Ze Xiong, Jason K. W. Lee, Yong Lin Kong, John S. Ho

**Affiliations:** 1 Institute for Health Innovation and Technology, National University of Singapore, Singapore, 117599 Singapore; 2 Department of Electrical and Computer Engineering, National University of Singapore, Singapore, 117583 Singapore; 3 Human Potential Translational Research Programme, Yong Loo Lin School of Medicine, National University of Singapore, Singapore, 119283 Singapore; 4 Department of Physiology, Yong Loo Lin School of Medicine, National University of Singapore, Singapore, 117593 Singapore; 5 The N.1 Institute for Health, National University of Singapore, Singapore, 117456 Singapore; 6 Global Asia Institute, National University of Singapore, Singapore, 119076 Singapore; 7 Institute for Digital Medicine, Yong Loo Lin School of Medicine, National University of Singapore, Singapore, 117456 Singapore; 8 Singapore Institute for Clinical Sciences, Agency for Science, Technology and Research (A*STAR), Singapore, 117609 Singapore; 9 Department of Mechanical Engineering, University of Utah, Salt Lake City, UT 84112 USA

**Keywords:** Electrical and electronic engineering, Sensors and biosensors, Soft materials, Electronic devices

## Abstract

Electronic textiles capable of sensing, powering, and communication can be used to non-intrusively monitor human health during daily life. However, achieving these functionalities with clothing is challenging because of limitations in the electronic performance, flexibility and robustness of the underlying materials, which must endure repeated mechanical, thermal and chemical stresses during daily use. Here, we demonstrate electronic textile systems with functionalities in near-field powering and communication created by digital embroidery of liquid metal fibers. Owing to the unique electrical and mechanical properties of the liquid metal fibers, these electronic textiles can conform to body surfaces and establish robust wireless connectivity with nearby wearable or implantable devices, even during strenuous exercise. By transferring optimized electromagnetic patterns onto clothing in this way, we demonstrate a washable electronic shirt that can be wirelessly powered by a smartphone and continuously monitor axillary temperature without interfering with daily activities.

## Abstract (web summary)

Electronic textiles should maintain performance against repeated mechanical, thermal and chemical stresses during daily activities. Here, authors demonstrate digital embroidery of robust liquid metal-based fibres with wireless functionalities.

## Introduction

Electronic textiles provide an attractive approach to seamlessly interface digital technology with the human body. By leveraging our inherent needs in clothing, electronic textiles can obviate the intrusiveness and risk often associated with implantable or epidermal electronics. In order for such clothing to function autonomously, textiles with a variety of technological capabilities are needed, including the ability to sense physiological signals, harvest energy, and wirelessly communicate data without causing discomfort or interfering with the user’s daily activities^1–6^. Such clothing can be used to establish a network of sensors, actuators, and displays around the body for applications ranging from health monitoring to human–computer interfaces^7^. However, achieving a seamless integration of electronics with clothing materials remains challenging. In contrast to the rigid and planar nature of conventionally manufactured electronics, textile is a porous structure composed of flexible materials that need to endure repeated mechanical, thermal and chemical stresses from daily activities as well as washing processes.

A broad range of innovative approaches has been explored to circumvent the inherent materials and geometrical incompatibilities. For example, button-sized electronics that integrate sensors, batteries, and wireless communication components that can directly attached to existing clothing have been developed^8,9^. Nevertheless, the functionalities, performance, and sensing opportunities available to such devices are constrained by the button-size footprint^10^. Textile functional system can be fabricated by printing/coating functional material such as conductive fillers into porous structure of the substrate textiles^2,11,12^. However, due to the mechanical mismatch, these approaches are prone to failures such as cracks and delamination. The infiltration also leads to discomfort, caused by the stiffening of the textile materials and blockage of porous structure that is needed for the transport of moisture and air. Hybrid strategies that combine both miniature electronic modules with large-area textile sensors can address the respective limitations of each approach^13,14^, but require solutions for powering and communication that do not intrude on daily activities.

Conductive threads can be digitally embroidered into clothing for wireless powering and communication using established computer-controlled textile manufacturing^15,16^. The seamless translation from digital design to fabrication can enable textiles with accurate reproduction of optimized wireless functional patterns^17,18^. Further, unlike coating and printing processes, the embroidered conductive threads can preserve the flexibility and permeability of the textile and leverage the existing manufacturing processes for scalable production. However, existing textile conductive materials have an inherent trade-off between mechanical and electrical performance. For example, metal-based threads have low  resistance per length (up to 2 Ω m^−1^) but also have a low yield strain (0.3% for copper), rendering it fragile under repeated bending^19–23^. Alternatively, carbon-based threads with elastic limits >5% strain have been developed, but currently have high resistance per length (>100 Ω m^−1^) that do not meet requirements for antennas and other radio-frequency components essential for wireless connectivity^24–26^.

In comparison to conductive textiles, liquid metal encapsulated with stretchable polymer, such as silicone rubber, can achieve high electrical conductivity and adaptation to mechanical changes^27–30^. These unique properties have been leveraged to create stretchable electromagnetic devices such as inductive coils^31–33^ and radiative antennas^34–36^ on elastomeric substrates. Recent works also demonstrate liquid metal fibers fabricated by filling liquid metal into polymer fibers for sensing and energy harvesting^37–40^. However, existing demonstrations have yet to fully leverage these properties to create textile threads compatible with digital embroidery or incorporate them into clothing to form autonomous systems.

Here, we demonstrate electronic textile systems with functionalities in near-field wireless power and communication created by digital embroidery of liquid metal fibers onto clothing. The liquid metal fibers are formed by infiltrating galinstan (Ga68.5/In21.5/Sn10) into perfluoroalkoxy alkane (PFA) tubing in a scalable process that yields fibers with high electrical and mechanical performance. Their compatibility with the digital embroidery process allows faithful reproduction of optimized radio-frequency patterns by leveraging established computer-controlled textile manufacturing^41^. We demonstrate the durability of the textiles when subjected to repeated stress and strain processes as well as multiple washing and drying cycles. We also show that these textiles can be used as a transmitter for wireless power and as an interface for near-field communication. In both contexts, the textiles facilitate robust and unobtrusive wireless connectivity with nearby devices including health sensors and a smartphone, even during strenuous exercise and daily activities. Such wirelessly enabled garments could be used to power bioelectronic implants in patients, detect diseases in high-risk individuals such as the elderly, and provide full-body tracking and feedback for athletes.

## Results

### Digital embroidery of liquid metal wireless systems

**Fig. 1:**
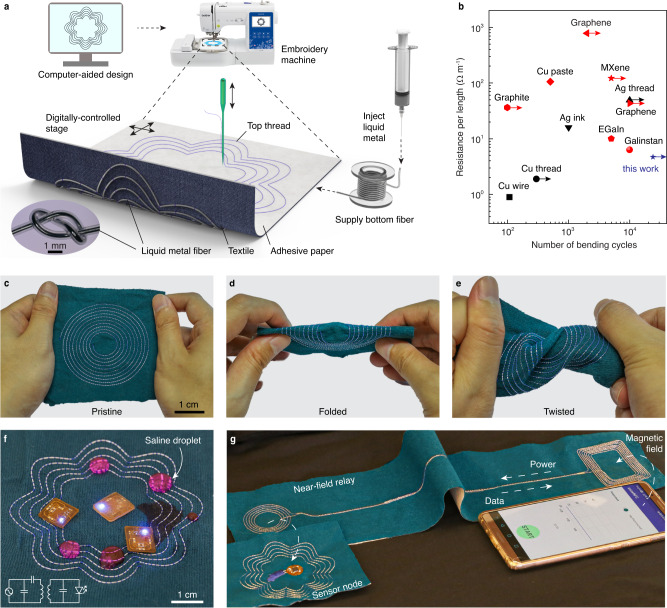
Digital embroidery of liquid metal wireless systems. **a** Illustration of the digital embroidery process. Liquid metal fibers consist of perfluoroalkoxy alkane tubing infiltrated with galinstan. **b** Electrical resistance per length and mechanical robustness of the liquid metal fibers compared to conductive materials used for textile (black color) or flexible (red color) electronics. Mechanical robustness is measured by the tested number of bending cycles. Arrows indicate materials have 10% resistance change happened after the maximum tested cycle. **c**–**e** Images of embroidered textiles subjected to folding and twisting. **f** Image of an embroidered spiral inductor wirelessly powering light-emitting devices in the presence of saline droplets. **g** Embroidered wireless system comprising a relay for long-range near-field communication between a smartphone and textile sensor node.

We used digital, computer-controlled embroidery of liquid metal fibers to accurately transfer computationally optimized electromagnetic designs onto existing clothing (Fig. 1a, Supplementary Note 1, Supplementary Movie 1). In contrast with alternative techniques to transfer patterns onto textiles, such as screen printing, digital embroidery is solvent-free, involves minimal modification of the substrate clothing, and is compatible with existing manufacturing techniques. The liquid metal fibers are fabricated by syringe injection of galinstan into the PFA tubing. The process is scalable as the peak pressure required is approximately twice the required pressure to fill a length of tubing with air (<109 kPa for 7.2 m length tubing, Supplementary Fig. 1). Of the many possible liquid metals, galinstan was selected owing to its excellent electrical conductivity (3.46 × 10^6^ S m^−1^ at 20 °C), liquid state at room temperature (melting temperature −19 °C) and low toxicity (Supplementary Note 2)^42^. PFA was selected for the tubing material for its excellent chemical, thermal, crack and stress resistance, low coefficient of friction, and high electrical insulation^43^ (Supplementary Table 1). The fibers have Young’s modulus of ~400 MPa and yield strain of 2.5%, which is mostly determined by the tubing material (Supplementary Table 2). The fiber’s gauge factor is ~1, and the change in resistance after stretching is <5% for 20% strain and after embroidery is <1% (Supplementary Fig. 2). The liquid metal fibers can achieve a fill ratio of ~95% (Supplementary Note 3) and electrical resistance per length of ~4.2 Ω m^−1^, which substantially exceeds existing conductive textiles with comparable mechanical robustness (>10^4^ bending cycles) (Fig. 1b, Supplementary Table 3). Textiles embroidered with the liquid metal fibers maintain robust electrical resistance (<1% variation) while being folded and twisted (Fig. 1c–e, Supplementary Movie 2), and are able to conform on curved surfaces (Supplementary Fig. 3). For the specific embroidery machine chosen in the proof of concept demonstration, other commercially available tubing materials such as polytetrafluoroethylene, vinyl, and polyurethane resulted in inaccurate reproduction of the pattern due to either low Young’s modulus or large tubing dimensions (Supplementary Table 2, Supplementary Fig. 4).

The digital embroidery process enables the fabrication of electronic textiles with functionalities in wireless power transfer and communication. Figure 1f shows an illustrative example in which the liquid metal pattern consists of an inductor optimized for near-field communication (NFC). Owing to the insulating properties of PFA, the textile can provide wireless power transfer to nearby battery-free devices even when exposed to or immersed in saline solution that simulates sweat (Fig. 1f, Supplementary Fig. 5). Figure 1g shows another design in which the liquid metal fibers form a near-field relay that extends the range of NFC. This textile enables connectivity to be established between a smartphone and a battery-free textile sensor located half a meter apart, provided that both are near to the inductive patterns. In this configuration, the wireless power transfer efficiency can be as high as 48%, compared to a maximum efficiency of 30% using commercially available conductive threads (~60 Ω m^−1^) (Supplementary Fig. 6). Beyond functioning as passive relays, the digitally embroidered textiles can also incorporate NFC chips to create clothing with system-level wireless sensing and data transfer capabilities. For example, the NFC antenna—which represents the largest component of the system—can be digitally embroidered onto the textile substrate, while the NFC chip and associated discrete components are assembled on a 1.3-cm diameter flexible printed circuit board (PCB) (Supplementary Fig. 7). The PCB is electrically connected to the liquid metal fibers using gold-coated metal sealing pins, secured to the textile by sewing, and encapsulated in electronic epoxy resin for water-proofing and strain isolation. The resulting textile system directly interfaces with smartphones via NFC without any connectors or additional external components, and remains fully functional even after multiple cycles of machine washing (Supplementary Fig. 8). Compared with the commercial NFC tag, such a fabric-based NFC tag relies on large antenna to increases the operating range from 4 to 7 cm in the plane of the textile, and from 7 to 10 cm in the vertical direction (Supplementary Fig. 9).

### Electromagnetic and mechanical performance

**Fig. 2:**
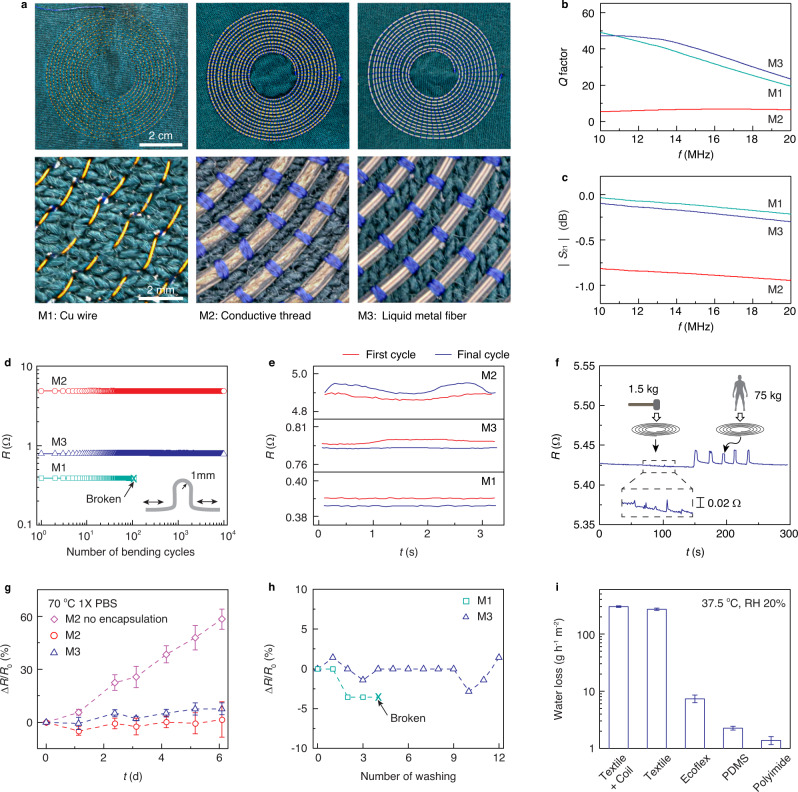
Electromagnetic and mechanical characterization. **a** Images of spiral inductors fabricated by embroidering copper wire (M1), conductive thread encapsulated with polyurethane (M2), and liquid metal fiber (M3). **b** Measured quality factor *Q* from embroidered spiral inductors. **c** Measured scattering parameter ∣*S*_21_∣ from embroidered 10-cm length transmission lines. **d**–**f** Resistance of the spiral inductors as a function of number of bending cycles (**d**), during the first and final bending cycle (**e**), and when compressed by the weight of a hammer or human adult (**f**). **g**, **h** Relative change in resistance when the spiral inductor is immersed in 70 °C 1× PBS (**g**) and subjected to cycles of machine washing (**h**). **i** Water loss through the spiral inductor, textile substrate, and conventional flexible substrates (Ecoflex, PDMS, and polyimide) at 37.5 °C and 20% relative humidity. Error bars show mean ± s.d. Source data are provided as a Source Data file.

We characterized the electromagnetic performance of the fabricated textiles using the spiral inductor pattern shown in Fig. 2a. The spiral inductor is optimized for wireless power transfer in the 13.56 MHz industrial, scientific, and medical band to a receiver comprising a 1.2-cm × 1.2-cm copper coil at a range of 1 cm (Supplementary Fig. 10). Figure 2b shows the measured quality factor *Q* of spiral inductors digitally embroidered on a textile substrate using three different kinds of threads: copper wires (AWG 34, ~160 μm in diameter), commercially available conductive threads with polyurethane encapsulation (~750 μm), and liquid metal fibers (~635 μm). The thread diameters are selected to have the highest value compatible with the embroidery process, which requires sufficiently low bending stiffness for machine manipulation. Textile spiral inductors based on liquid metal fibers achieved the highest quality factor of *Q* = 44.4 at 13.56 MHz, which exceeds that of the copper wires *Q* = 39.9 (due to the larger embroidery-compatible diameter), and is about 7 times that of conductive threads *Q* = 6.5 (Fig. 2b). To measure radio-frequency losses, we also used the digital-embroidery process to fabricate parallel-wire transmission lines (Supplementary Fig. 11). Measurements of the scattering parameter *S*_21_ for a 10-cm length transmission line show that liquid metal fibers achieve transmission efficiencies that are comparable to copper wire (~0.02 dB cm^−1^) at 13.56 MHz, and significantly higher than conductive thread (~0.1 dB cm^−1^) (Fig. 2c). At frequencies higher than 300 MHz, the loss increases significantly to about 1.5 dB cm^−1^ owing to the skin effect (Supplementary Fig. 11); this loss may be mitigated by using multichannel tubing to increase the surface area.

We next performed mechanical tests on the electronic textiles to assess their robustness to daily wear. Cyclic bending tests show that the embroidered spiral inductors exhibit <1% change in the electrical resistance over 10^4^ cycles, which provides comparable durability compared to the commercially available conductive threads (Fig. 2d). In contrast, copper wire breaks after 108 cycles owing to its low yield strain (~0.5%) relative to the liquid metal fibers (~2.5%) (Supplementary Table 2). The resistance of the textiles also remained constant within each bending cycle (Fig. 2e) and even when compressed by a 1.5-kg hammer or a 75-kg human (<1% variation, Fig. 2f). For comparison, conductors fabricated by filling liquid metal into soft channels such as polydimethylsiloxane (PDMS) and Ecoflex generally exhibit significant change (>100% variation) in resistance under similar compressed deformations^44,45^, which results in undesirable variations in the electromagnetic performance.

We also evaluated durability of the electronic textiles exposed to thermal changes, moisture, biofluids, and washing cycles. The embroidered spiral inductors remained functional when submerged in 70 °C 1× phosphate-buffered saline (PBS) bath for 150 h (<10% change in resistance, Fig. 2g) and over 10 h of machine washing (12 cycles, <3% change in resistance, Fig. 2h, Supplementary Movie 3). Specially, over 24,000 cycles of bending in 50 °C 1× PBS to simulate daily wear conditions, the textiles exhibited <2% change in resistance (Supplementary Fig. 12). In contrast, silver-plated conductive threads without an encapsulating layer exhibit ~50% increase in resistance in the hot PBS over the same duration (Fig. 2g). The same pattern reproduced using digital embroidery of copper wires were electrically broken after four wash cycles (Fig. 2h) and delaminated from the textile substrate after 10 h of machine washing (Supplementary Fig. 13). Furthermore, the embroidery process maintains the porous structure of the substrate such that the water permeability of the spiral inductor is the same as the underlying textile. The permeability of the embroidered textiles is as high as 300 ± 7.7 g h^−1^ m^−2^, which is more than 40 times higher than that of soft substrates such as PDMS, Ecoflex, and polyimide commonly used in flexible electronics (Fig. 2i).

### Embroidered wireless powering systems

**Fig. 3:**
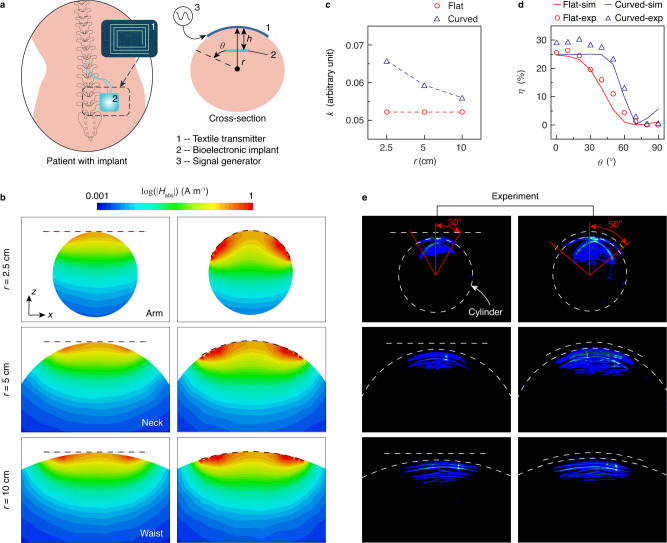
Textile wireless power transmitters for implants. **a** Illustration of a textile transmitter being used to wirelessly power a bioelectronic implant. The cross-section view shows the radius of curvature *r*, device depth *h*, and alignment angle *θ*. The transmitter is connected to a signal generator, which can be integrated in a wearable form factor. **b** Cross-section view of simulated magnetic field ∣*H*_abs_∣ generated by a flat and conformal transmitter placed on arm, neck and waist. Dotted black lines show the transmitter position. **c** Coupling coefficient *k* between flat or conformal transmitters and a receiver placed at *h* = 1 cm, *θ* = 0°. **d** Corresponding wireless power transfer efficiency *η* as a function of *θ* for *r* = 2.5 cm based on simulation and experimental measurement. **e** Long-exposure images of the body phantoms (1× PBS inside acrylic cylinders with *r* = 2.5, 5 and 10 cm) where position of the receiver is scanned inside. The receiver is configured with a blue light-emitting diode (LED) that activates when sufficient power is transferred. The input power of the liquid metal textile transmitter is 20 dBm. Source data are provided as a Source Data file.

We used the digital embroidery process to create textile transmitters for wireless power transfer to battery-free bioelectronic implants (Fig. 3a, Supplementary Table 4). Such implants are being clinically used to treat a wide range of disorders including pain and urinary incontinence, but currently rely on rigid transmitters that need to be either strapped against the body or held over the implanted device^46,47^. We investigated the use of a conformal textile transmitter to wirelessly power a bioelectronic device with dimensions of 1.2 cm × 1.2 cm. The device is placed within a body phantom comprising an acrylic cylinder filled with 1× PBS with radius of 2.5, 5, and 10 cm in order to model the arm, neck, and waist. Full-wave simulations show that the ability of textile transmitter to conform to curved surfaces increases the penetration of the magnetic field into the body relative to a planar transmitter (Fig. 3b). Experimental measurement of the coupling coefficient when the receiver is placed at 1-cm depth in the 2.5-cm radius body phantom shows that the conformal transmitter achieves *k* = 6.5 × 10^−2^ compared to *k* = 5.2 × 10^−2^ for the rigid transmitter (Fig. 3c). In addition, the conformal transmitter provides transfer efficiency *η* > 20% within a ±50° angular region, whereas the operating region is limited to ±30° when the rigid transmitter is used (Fig. 3d). We also mapped the efficient powering area by scanning the position of a receiver configured with a blue light-emitting diode (LED) inside cylinder phantoms (Supplementary Fig. 14). Long-exposure images show that the conformal transmitter provides greater wireless power transfer coverage compared to the rigid transmitter for the 2.5 and 5-cm radius phantom (Fig. 3e). For the 10-cm radius phantom, the coverage provided by both transmitters is comparable owing to the negligible curvature of the surface.

**Fig. 4:**
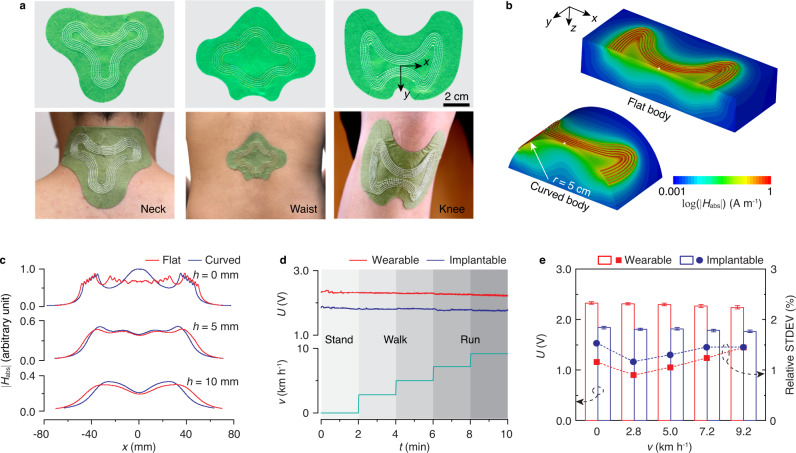
Conformal textile skin patches. **a** Images of skin patches fabricated using digital embroidery of liquid metal fibers attached to the neck, waist, and knee. The skin patches are used as wearable transmitters connected with a signal generator for wireless powering of the underneath wearable and implantable devices during daily activities. **b** Simulated magnetic field ∣*H*_abs_∣ on a flat and curved (5-cm radius) body phantom. The cutting plane is set to the center of the skin patch (white dot). **c** Field profiles along the cutting plane at varying depth *h*. **d** Received voltage *U* of wearable and implantable device, wirelessly powered by the skin patch, during physical activities with speed *v* increasing from 0, 2.8, 5, 7.2, to 9.2 km h^−1^, indicated by the shadows. The input power of the skin patch is 20 dBm. **e** Comparison of *U* and its relative standard deviation (STDEV) during different activities. Error bars show mean ± s.d. Source data are provided as a Source Data file.

The digital embroidery process allows versatile fabrication of textile transmitters with irregular shapes and patterns. Figure 4a shows three patch transmitters with shapes optimized for attachment onto various body surfaces, including the neck, waist, and knee. Full-wave simulations show that the designs operating at 13.56 MHz provide a magnetic field distribution compatible with efficient power transfer at 1-cm distance on both flat and curved (5-cm radius) body surfaces (Fig. 4b, c, Supplementary Fig. 15). We experimentally evaluated the variability of wireless power transfer by measuring the rectified voltage at the receiver (copper coil with dimensions 1.2-cm × 1.2-cm) when the textile transmitter operated at an input power of 20 dBm (Supplementary Fig. 16). The receiver is placed in both a wearable and implantable configuration, in which the device is either attached on the skin under the transmitter or placed under a 1-cm-thick human tissue phantom (see the “Methods” section). Figure 4d shows the received voltage over 10 min as the subject stands, walks, and runs on a treadmill as the speed is increased in a stepwise manner. For the wearable configuration, the relative standard deviation of the received voltage is <1.45% within each 2-min interval. The average received voltage also remains relatively stable between each interval, decreasing from ~2.33 V when standing still to ~2.24 V when running at 9.2 km h^−1^ (Fig. 4e). Similarly, for the implantable configuration, the relative standard deviation is <1.53% and the received voltage decreases from ~1.84 to ~1.76 V. The average received voltage is lower due to the intervening tissue phantom and could be enhanced for specific application by using a higher input power, adding a voltage regulator or modifying the rectifier circuit.

### Continuous thermal monitoring with battery-free textiles

**Fig. 5:**
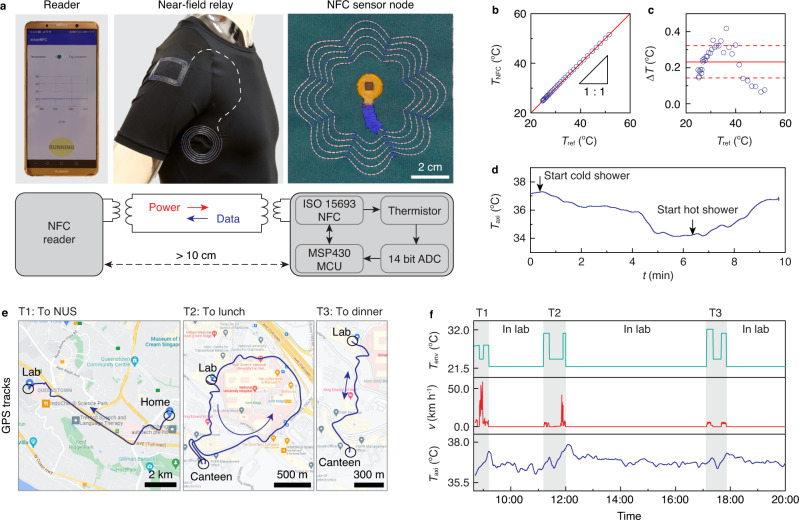
Continuous axillary temperature monitoring. **a** Textile thermal monitoring system comprising a sensor node connected with a smartphone reader via a near-field relay integrated on a shirt. The top row shows images of the system components while the bottom row shows the block diagram. The distance between the reader and sensor node is more than 10 cm apart. **b** and **c** Calibration of the measured temperature *T*_NFC_ with a reference thermometer *T*_ref_. Δ*T* = *T*_ref_−*T*_NFC_ is the temperature difference. The red solid line and dash lines in (**c**) show mean ± s.d. **d** Axillary temperature *T*_axi_ measured in a shower. The cold shower used ~25 °C water while the hot shower used ~40 °C water. **e** Map of the GPS position of the subject. **f** Axillary temperature *T*_axi_ streamed by the wireless system over a day. Velocity *v* of the subject and environment temperature *T*_env_ obtained from the smartphone are plotted for reference. Source data are provided as a Source Data file.

To demonstrate the utility of our textiles for health sensing applications, we developed a wireless and battery-free shirt that provides continuous monitoring of axillary temperature, which is an important indicator of exhaustion, performance, and various health conditions^48^. This shirt wirelessly streams data from a digitally embroidered thermal sensor placed on the underarm to a custom application on a smartphone worn on the arm via NFC at 13.56 MHz (Fig. 5a, Supplementary Table 4). The offset of the thermal sensor is 0.24 ± 0.075 °C when the target temperature ranges from ~25 to ~55 °C compared to a thermometer (Fig. 5b, c). Continuous streaming of thermal data in a shower illustrates the ability of the shirt to operate in wet environments and establish connectivity with a smartphone through a water-proofing pouch (Fig. 5d). The sampling rate of the sensor is 8 Hz (14 bit) and can be further increased up to 12 Hz for more demanding applications such as respiration sensing and gait monitoring. The use of the textiles does not affect the sampling rate, which is determined by the underlying NFC protocol (Supplementary Fig. 17).

Continuous monitoring was performed on a single male subject over 12 h of daytime activities. The location of the subject over this period was tracked using a smartphone GPS application. The GPS trace shows three major changes in location: commuting from home to campus (T1), going from the lab to lunch (T2), and going from the lab to dinner (T3) (Fig. 5e). Figure 5f shows the corresponding axillary temperature *T*_axi_ acquired from the shirt alongside the velocity *v* and environmental temperature *T*_env_. When the subject is in the lab, indicated by the stationary *v* trace and an indoor environmental temperature *T*_env_ = 22 °C, continuous thermal monitoring reveals that *T*_axi_ fluctuates within the range 36.5 ± 1 °C. When the subject is outdoors, however, the data trace indicates that *T*_axi_ increases above 37 °C, owing to the warm outdoor climate *T*_env_ > 28 °C. Furthermore, *T*_axi_ increases with the duration of outdoor activity, which can be attributed to thermal due to exercise (walking at *v* ≈ 5.5 km h^−1^), and returns to baseline levels after the subject enters an indoor environment. Data was acquired by the smartphone in real-time at a 0.1-Hz sampling rate without any loss of connectivity over the 12-h duration. In addition, the shirt with the embroidered NFC module did not exhibit any degradation of function after 10 h of machine washing (Supplementary Fig. 8). These results demonstrate that digital embroidery of liquid metal fibers can fabricate clothing with broad utility for health monitoring during daily activities.

## Discussion

We have demonstrated digital embroidery of liquid metal fibers to create electronic textiles with near-field wireless functionalities in powering and communication. Owing to the unique properties of the liquid metal fibers, these electronic textiles can possess both high electrical conductance and mechanical performance, allowing them to establish wireless connectivity without compromising the durability or breathability of the underlying clothing. Importantly, the compatibility with the digital embroidery process enables the faithful and scalable reproduction of optimized near-field inductive patterns design with full-wave electromagnetic simulations. Demonstrations of a wireless power transmitter and a thermal sensing garment show that our approach can provide robust wireless connectivity even during daily activities.

Future work should address the long-term durability of the electronic textiles in specific applications. For instance, prolonged outdoor use may expose the textiles to ultraviolet light, chemicals, abrasion, and other forms of stress that are not present in standard tests^49,50^. The use of alternative tubing materials, such as fluorinated ethylene propylene or nylon, can be explored to enhance the ability of the fibers to resist such stressors^51^. Increasing the hardness of the fibers, such as by using a liquid–solid metal composite core or high strength tubing material, can allow the fibers to withstand perforation by sharp tools, although at the expense of the flexibility of the textile. Significantly, the future integration of additional electronic subsystems can further expand the functionality of the electronic textiles. For example, an integrated radio-frequency module can be used to operate the textile wireless power transmitters without external connections^52^, allowing the system to function autonomously with intermittent wireless charging. Leveraging the versatility of digital embroidery, the radio-frequency patterns transferred onto the textiles can also be adapted for other wireless protocols, such as Qi for wireless charging^53,54^ and Bluetooth for high-data-rate communication^55,56^. Electronic textiles with these and other wireless capabilities can be used for continuous and unobtrusive monitoring in a broad range of applications, especially healthcare, sports and defense industries.

## Methods

### Fabrication process

Spiral inductors were designed by using electromagnetic simulation software (CST Microwave Studio, Dassault Systems) and converted to a stitch trajectory by a commercial software (PE-DESIGN 10, Brother). Fabrication used a digital embroidery machine (NV180, Brother) fed with liquid metal fibers produced by filling galinstan into perfluoroalkoxy alkane (PFA) tubing. The current embroidery machine has a working area of 10 cm × 10 cm for digital control which can be arbitrarily extended using manual control. Further, an embroidery area with meter scale can be achieved by using other machines (JCZA 0109-550 (700), ZSK technical embroidery systems). A gold-coated metal pin was inserted into the tubing for both sealing and interconnection. Flexible print circuit boards were designed with two tails (1-cm in length, 0.3-mm in width) which were inserted into liquid metal fibers and sealed by a metal pin to secure the mechanical and electrical connection.

### Textile characterization

Transmission lines (10-cm length) were characterized by the scattering parameter *S*_21_ and spiral inductor (6-cm diameter, 1.5-mm gap, and 13 turns) by the impedance parameter *Z*_11_ measured using a vector network analyzer (Keysight, FiledFox N9914A) and coaxial cables (SMA-SMA, 50 Ω, Amphenol). The frequency range used was 10–20 MHz and the quality factor was calculated as ${{{{{{{\rm{Im}}}}}}}}({Z}_{11})/{{{{{{{\rm{Re}}}}}}}}({Z}_{11})$.

Bending tests used a linear stage with the sample clamped at both ends. The distance between two ends was varied from 13 to 3 mm at a speed of 5 mm s^−1^, which bends the thread to a minimum radius of ~1 mm. The thread was bent repeatedly up to 10,000 cycles while the electrical resistance was recorded continuously by a multimeter (Keithley 2450, Tektronix). The tensile test was carried out using the same set-up by stretching the thread at a speed of 1 mm s^−1^ until breaking. The thermal test immersed conductive materials with a length of ~10 cm in 1× PBS, which was stored in a 70 °C chamber. The materials were taken out of the chamber and the electrical resistance measured at room temperature.

Washing tests followed the ISO 6330 standard for domestic washing and drying^57^. The electronic textile was combined with clothing in a household washing machine (MT725B, Midea) to form a 2.3 kg load. An anti-bacterial powder detergent (DYNAMO, The Procter & Gamble Company) was added and the washing program set to a standard 50-min washing, rinsing, and spinning cycle. Functionality of the electronic textiles after washing was verified by measuring the end-to-end resistance using a multimeter. Textile permeability tests were performed by sealing a glass bottle filled with 100-g water with the textile sample. The bottles were placed in a chamber with 37.5 °C and 20% relative humidity. Water loss was calculated by measuring the remaining mass.

### Numerical simulation

Electromagnetic simulations used CST Microwave Studio (Dassault Systems) using the frequency domain solver to compute the field distributions at 13.56 MHz. The receiver was modeled using a copper planar inductor with dimensions of 10.4 mm × 11.9 mm, 0.127-mm track width, 0.254-mm gap and 5 turns. The distance between the transmitter and receiver was set at 1 cm unless otherwise stated. For the implantable configuration, tissue was modeled by a dielectric permittivity of 78.4 and conductivity of 0.93 S m^−1^.

### Wireless measurements

Wireless power experiments used acrylic cylindrical containers with radii of 2.5, 5, and 10 cm filled with 1× PBS. The transmitter was a rectangle spiral liquid metal antenna (37 mm × 52 mm, 3-mm gap, and 4 turns) and the receiver the copper planar inductor previously used in numerical simulations. A blue LED was connected to the terminals of the receiver inductor for visualization. Both the transmitter and receiver were tuned to resonate at 13.56 MHz using matching networks. While the transmitter was driven by a signal generator with a continuous sinusoidal wave (input power of 20 dBm at 13.56 MHz), the receiver was moved inside the cylinder, and the LED recorded by a camera (Canon 6D) using a 30-s exposure.

On-body wireless power experiments used a transmitter fabricated by digital embroidery of a fabric-based skin patch. The patch is a commercially available disposable sticker (Wormwood sticker, Ai Yi Shi) that has an adhesive layer on one side. The result is an ergonomic patch with a 5 turn, 3-mm gap inductive pattern that can be conformally placed on various curved body surfaces. The receiver used the same copper antenna described in previous paragraph, which was matched to resonant at 13.56 MHz, and was either directly attached to the skin under the patch or placed under a 1-cm-thick human tissue phantom (1% Agarose gel in 1× PBS) to mimic the wearable and implanted configurations. The human subject carried out activities on a treadmill with speed increasing from 0, 2.8, 5, 7.2, to 9.2 km h^−1^, with each speed maintained for 2 min. During the whole process, the patch was driven by a signal generator with a continuous sinusoidal wave (20-dBm power, 13.56-MHz frequency), while the received voltage after rectification was measured using an oscilloscope (Picoscope 5444D, Pico Technology).

### Temperature sensing

Continuous axillary temperature monitoring was carried out on a human subject wearing an athletic shirt integrated with a near-field relay. The relay was composed of a rectangular hub (7 cm × 5 cm dimension, 5 turns and 2-mm wire gap) at the sleeve and a circular terminal (8-cm outer radius, 5 turns and 3-mm wire gap) at the armpit. A NFC-compatible smartphone and a temperature sensor node were respectively placed proximity to the hub and terminal to establish wireless interconnection. The temperature sensor node was composed of a liquid metal antenna (8-cm diameter, 3-mm wire gap, and 5 turns) and a 1.3-cm diameter PCB assembled all electronic components, including a NFC chip (RF430FRL152H, Texas Instruments) and an integrated NTC thermistor (ERTJ1VS104A, Panasonic Co. Ltd.). GPS tracks and speed of the human subject were recorded by the smartphone. Environment temperature was recorded by a thermometer (HH506RA, Omega) connected with a thermocouple (K-type). The temperature sensor node was calibrated against the thermometer with the thermocouple was closely placed (<1 mm distance) around the thermistor.

All experiments complied with a protocol approved by the National University of Singapore Institutional Review Board (NUS-IRB-2021-635). All subjects were volunteers, were informed of risks and benefits, and provided informed consent.

## Supplementary information

Supplementary Information

Description of Additional Supplementary Files

Supplementary Movie 1

Supplementary Movie 2

Supplementary Movie 3

## Data Availability

Source data for main figures are provided with this paper. Other raw and analyzed datasets generated during the study are available for research purposes from the corresponding authors on reasonable request. Source data are provided with this paper.

## Source data

Source Data
