# Supplementary material for: Digitally-embroidered liquid metal electronic textiles for wearable wireless systems
Source: Nat Commun. 2022 Apr 21;13:2190. doi: 10.1038/s41467-022-29859-4 (PMC9023486; doi:10.1038/s41467-022-29859-4)
Supplement: Supplementary file 1 — Supplementary Information [file 41467_2022_29859_MOESM1_ESM.pdf]

Supplementary Information for

Digitally-embroidered liquid metal electronic textiles for wearable wireless systems

R. Lin et.al

Supplementary Note 1. Comments on conductive textile materials.

Conductive textile material is one of the most important components of electronic textiles that have challenging competing requirements to satisfy. In addition to achieving high electrical conductivity, conductive textiles must be sufficiently durable and comfortable for daily use. In general, the current approach can be categorized based on the conductive filler materials (metal, carbon) and their configuration (filament, polymer plated metal, composite) as described below.

Metallic threads, include threads consists of bundles of metal filaments (*e.g.*, ELEKTRISOLA) and metal filaments covered by or twisted with polymer filaments (*e.g.*, IBTECH Innovations), generally achieve the highest electrical conductivity among textile conductive materials. The composition of multiple filaments also enables high electrical conductivity even at radio-frequency applications. The high Young's modulus requires small dimensions to enable metallic threads flexibility for both wearing comfortability and compatibility with conventional textile fabrication techniques at the cost of lowing overall electrical conductance. Due to the low elastic limit, folding and washing could easily crumple the metal threads. Even though innovations in material (such as using alloy) and structure (twisted with polymer filament) can relieve the sensitivity against deformation, the durability is still inherently limited by the low yield strain of metal, which are delicate against repeatably folding or washing.

Polymer-plated/coated metal conductive threads are composed of filaments with a high-strength polymer core and a conductive metal outer layer (*e.g.,* Syscom Advanced Materials, Shieldex). Such conductive threads are developed to have the attributes of existing non-coated threads to be compatible with processes such as twisting, warp- and weft-knitting, embroidering, weaving, and spinning. However, current commercially available conductive threads are still limited by the trade-off between mechanical robustness and electrical conductance. The brittle nature of the metallic coating layer can be cracked or even disintegrated during the washing process. The metallic coating can also be oxidized, sulfided, or corroded when exposed to heat, moisture, sweat, and other chemicals during daily activity.

Metallic composites are composed of metallic filler inside the polymer matrix. By engineering the conductive and matrix material, metallic composites can achieve

higher flexibility and stretchability but often at the cost of electrical conductivity. Further, the electrical conductance is sensitive to deformation as its conductance is dictated by the percolation network formed by the metallic element. The fabrication of the metallic also requires the usage of solvent to disperse conductive filler inside the matrix material. While the step is needed to achieve printable viscosity in the fabrication process, the solvent may dissolve the textile substrate and affect the comfort of the textile. The dissolved substrate can block the porous structure needed for the transport of moisture and air. Development of metallic composites in the fiber shape may avoid these problems and facilitate scalable integration on textile using conventional textile techniques.

Carbon-based conductive textile materials have similar configurations as the metallic conductive textile materials, but use carbon nanomaterials instead of metal as conductive components. In comparison with the metallic counterparts, carbon-based textile conductive material could achieve higher flexibility, stretchability, lightweight and stability in high moisture environments. However, carbon material generally has much lower electrical conductivity and has limited efficiency in power transmission.

In contrast to current conductive textile materials, **the liquid metal fibers** can achieve simultaneously high mechanical and electrical properties desirable for a conductive textile without compromising one over another. The polymer sheath can impart the needed mechanical properties, and the liquid metal core can provide the needed electrical conductivity. As a proof of concept, we demonstrate that this is achievable by filling PFA tubing with galinstan to create an embroidery-compatible liquid metal fiber. The fibers achieve electrical conductance close to copper wires (with a smaller diameter) and outstanding durability against mechanical, thermal, moisture and chemical stress during fabrication process, daily wearing and washing. In addition, we anticipate that the electrical and mechanical properties of the liquid metal fibers can be further optimized with the development of core and shell materials.

Supplementary Note 2. Biocompatibility of liquid metal

Liquid metal is a unique class of low viscosity fluid that possesses the high electrical and thermal conductivity of a metal. Mercury is one of the most well-known liquid metals, which have been used in thermometers and barometers since 1643 despite its toxicity. In contrast to mercury, Gallium and its alloys have been demonstrated to have low toxicity and have been used in biomedical applications. Previous works have explored the usage of gallium alloys as carriers for drug delivery, contrast agents for molecular imaging, and incorporated to implantable devices such as bioelectrodes and nerve connectors, and are also used as wearable devices including antenna, heater, and sensors[S1-S4].

The zero-vapor pressure at room temperature enables handling without concern for inhalation. The negligible solubility in water prevents feasible entering into the blood stream[S1, S3]. The cytotoxicity of the gallium alloys based liquid metal has also been demonstrated on several kinds of cells[S4], such as human epidermal HaCaT cells[S5], human melanoma C8161 cells[S5], HeLa cells[S6], and even neurons, which are notoriously sensitive[S7]. Earlier work has demonstrated that liquid metal particles can be safely introduced into rodents[S4, S8]. Tissue or organ damages, or significant pathological changes or inflammatory responses have not been observed. Nevertheless, more studies are required to determine the long-term safety attributes of liquid metal such as the effect size, composition, and degree of mechanical agitation[S9].

Supplementary Note 3. Effect of galinstan saturation on electrical conductance.

Saturation of Galinstan inside the tubing can be defined as the filled ratio, the ratio between galinstan volume and tubing cavity volume. We assume that galinstan distributes as a cylinder shape inside the tubing. In the DC region, the electrical resistance of the liquid metal fiber can be calculated from

$$R_{DC} = \frac{l}{\sigma A} = \frac{l^2}{\sigma V}$$

where l, A, V is the length, cross-section area and volume of galinstan, $\sigma = 3.46 \times 10^6$ S m^{-1} at 20 ^{o}C is the electrical conductivity of galinstan. Thus, R_{DC} increases reversely with the filled ratio of galinstan inside the tubing. In the AC region, considering the skin effect, the electrical resistance is

$$R_{AC} \approx \frac{l}{\sigma \pi d \delta} \approx \frac{l^{3/2}}{\sigma \delta \sqrt{4\pi V}}$$

where δ is the skin depth, and d is the cylinder diameter of galinstan. Thus, R_{AC} increases reversely with the square root of the volume of galinstan inside the tubing. In this manuscript, the filled ratio of galinstan inside the tubing is ~ 94.6 % estimated by comparing the theoretical and measured DC electrical conductance, which is 3.96 Ω m^{-1} (PFA tubing have 0.305 mm inner diameter) and ~ 4.17 Ω m^{-1}, respectively.”

Supplementary Table 1. Comparison of core-shell liquid metal fibers.

Core material	Shell material	Inner diameter (μm)	Outer diameter (μm)	Textile integration approach	Washability (hours)	Fatigue testing (cycles)	Application	Ref.
Galinstan	PFA	305	635	Embroider	10	24000	Wireless interconnection	This work
Galinstan	Geniomer	200	930	Weave	0.67	-	Sensing, energy harvesting	[S19]
Galinstan	SEBS	200	1500	-	-	-	Sensing	[S20]
Galinstan	TPU	600	1000	-	-	200	Sensing	[S21]
EGaIn	Dragon skin	800	2000	-	-	-	Thermo-/mechano-chromic	[S22]
EGaIn	PDMS	100	150	-	4	500	Sensing	[S23-S24]
EGaIn	PDMS	550-750	850-1050	-	-	3500	Sensing	[S25]
EGaIn	SEBS	360-1030	440-1370	-	-	-	Sensing, energy harvesting	[S26-S28]
EGaIn	SEBS	600	800	-	-	-	Sensing	[S29]
EGaIn	Tygon	244	704	Sew	-	1500	Sensing	[S30]
EGaIn-PVDF-HFP-TFE	PVDF-HFP-TFE	230	270	Weave	-	-	Sensing, energy harvesting	[S31]
Ga-In-Sn-Zn	PU	300	340	-	-	-	Sensing, energy harvesting	[S32]

PFA, Perfluoroalkoxy alkane. SEBS, Styrene-ethylene-butylene-styrene. TPU, Thermoplastic Polyurethane. PDMS, Polydimethylsiloxane. PVDF-HFP-TFE, Poly (vinylidene fluoride-hexafluoropropylene-tetrafluoroethylene). PU, Polyurethane.

Supplementary Table 2. Dimensions and mechanical properties of conductive material and flexible tubing.

Material	Outer diameter (mm)	Inner diameter (mm)	Young's modulus (MPa)	Yield strain (%)	Embroidery compatibility
Liquid metal fiber	0.635	0.305	399 ± 32.6	2.5 ± 0.10	✓
Copper wire	0.205	0	31180 ± 4086	0.5 ± 0.03	✓
Conductive thread	0.726	0	281 ± 24.3	>15	✓
Perfluoroalkoxy alkane (PFA)	0.635	0.305	433 ± 27.5	2.4 ± 0.17	✓
Polytetrafluoroethylene (PTFE)	0.762	0.304	1037 ± 21.6	1.9 ± 0.24	✗
Vinyl	0.64	0.28	86.6 ± 3.78	3.2 ± 0.13	✗
Polyurethane (PU)	0.635	0.305	16.4 ± 0.43	15.3 ± 1.19	✗

Supplementary Table 3. Comparison of conductive materials for wireless applications based on electrical resistance and mechanical robustness.

Conductive material	Substrate material	Resistance (Ω m^{-1})	Dimension [a] (μm)	Tested cycles	Relative change [b]	Radius [c] (mm)	Ref.
Liquid metal fiber	Textile	4.2	305	24000	< 1%	1	This work
Copper wire	Textile	0.9	205	108	break	1	This work
Conductive thread	Textile	48.9	370	10000	< 1%	1	This work
Elektrisola E-threads	Textile	1.9	120	300	-	-	[S10]
Silver ink	Textile	16	1000 × 146	1000	5% ~ 10%	3	[S11]
Copper paste	PET	105	1000 × 100	500	> 800%	5	[S12]
Graphene	Paper	780	1000 × 35	2000	5%	-	[S13]
Graphene	PEN	43	1000 × 55	10000	< 5%	45	[S14]
Graphite	-	36	1000 × 25	100	-	-	[S15]
Mxene	PET	121	1000 × 5.5	5000	< 1%	5	[S16]
EGaIn	PDMS	10	289 × 80	5000	10%	8.1	[S17]
Galinstan	PVA	6.4	300 × 150	10000	< 3%	0.5	[S18]

PET, Polyethylene terephthalate. PEN, Polyethylene naphthalate. PDMS, Polydimethylsiloxane. PVA, Polyvinyl acetate.
[a] Characteristic dimension for conductive material in fiber/thread and plane format is radius and width × thickness, respectively.
[b] Relative change is the relative resistance change through the whole bending test against primary resistance.
[c] Radius is the minimum bending radius during bending test.

Supplementary Table 4. Configuration of wireless systems.

**Figure**	**Transmitter**	**Relay**	**Receiver**
Fig.1f, 3, 4,<br>Supplementary Fig.3, 4, 9, 14, 15, 16	Liquid metal textile	-	Copper coil
Fig.1g, 5<br>Supplementary Fig.17b	Smartphone	Liquid metal textile	Liquid metal textile
Supplementary Fig.5	Liquid metal textile	Liquid metal textile	Liquid metal textile
Supplementary Fig.7, 8,17a	Smartphone or NFC reader	-	Liquid metal textile or copper coil

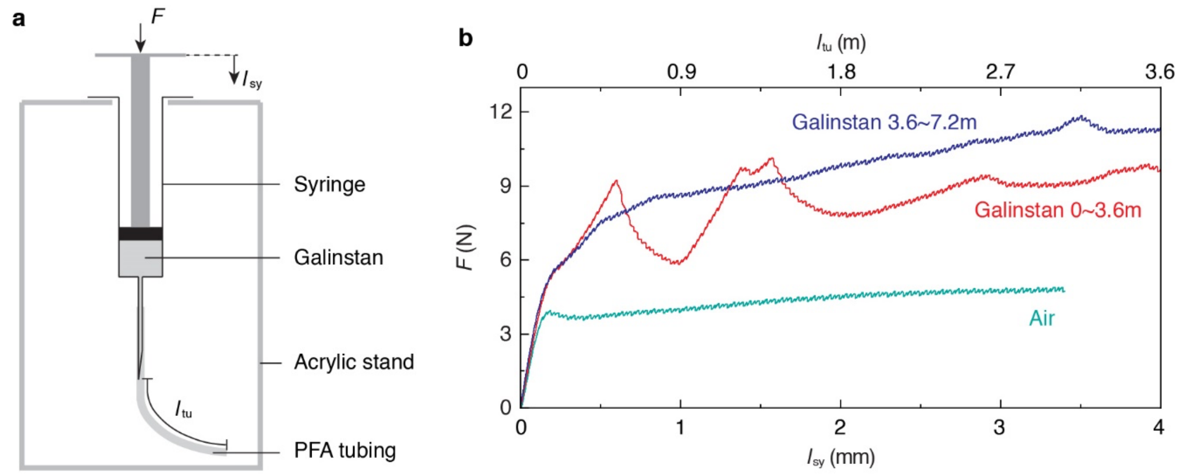

Supplementary Figure 1. Injecting force. a, Schematic illustration of the experimental setup for measuring injecting force. A 3-mL syringe (9-mm inner diameter) connected with a Gauge 27 needle is used to inject air/galinstan into the PFA tubing with an inner diameter of 0.3 mm. The injecting force *F* is continuously measured by a force sensor versus the displacement of the plunger l_{sy} and the filled length l_{tu} while the plunger is pushed by a linear stage at a constant speed of 0.01 mm s^{-1}. Correspondingly, galinstan is injected into the tubing at a speed of 9 mm s^{-1}. **b**, The injection force (*F*) against displacement l_{sy} and l_{tu} for air or galinstan injection into the PFA tubing. Galinstan is injected into the tubing in two stages. At stage one, the tubing is filled from 0 to 3.6 m when the plunger is pushed to 4 mm. At stage two, the plunger is retrieved to release pressure and pushed to another 4 mm, while the tubing is filled from 3.6 to 7.2 m.

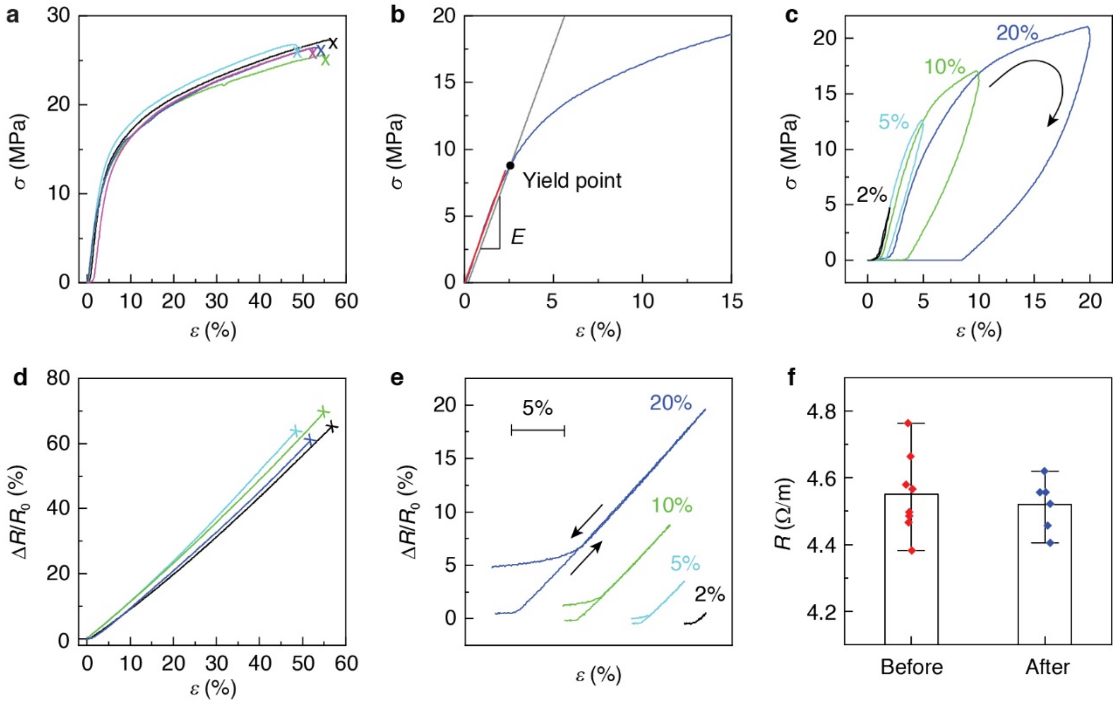

Supplementary Figure 2. Mechanical and electrical characterization of liquid metal fibers. a, Stress (*σ*) - strain (*ε*) curves of liquid metal fibers. The pulling speed of 1 % s^{-1} (strain per second) is used in all the experiments. **b**, Zoom-in of the *σ*-*ε* curve. Redline shows linear fit within the elastic range (Young's modulus *E* is estimated from the slope). Green line is a 0.2 % shift of the red line along the *x*-axis. The yield point is the intersection of the green line with the *σ*-*ε* curve. **c**, Stress responses of the liquid metal fibers with cycling strain varying from 2 %, 5 %, 10 % to 20 %. **d-e**, Relative electrical resistance changes $\Delta R/R_0$ of the liquid metals fibers against strain until break (**d**) and against cycling strain varying from 2 %, 5 %, 10 % to 20 % (**e**). **f**, Electrical conductance *R* of the liquid metal fibers before and after embroidered on textiles. The error bars show mean ± s.d. (n > 6 samples).

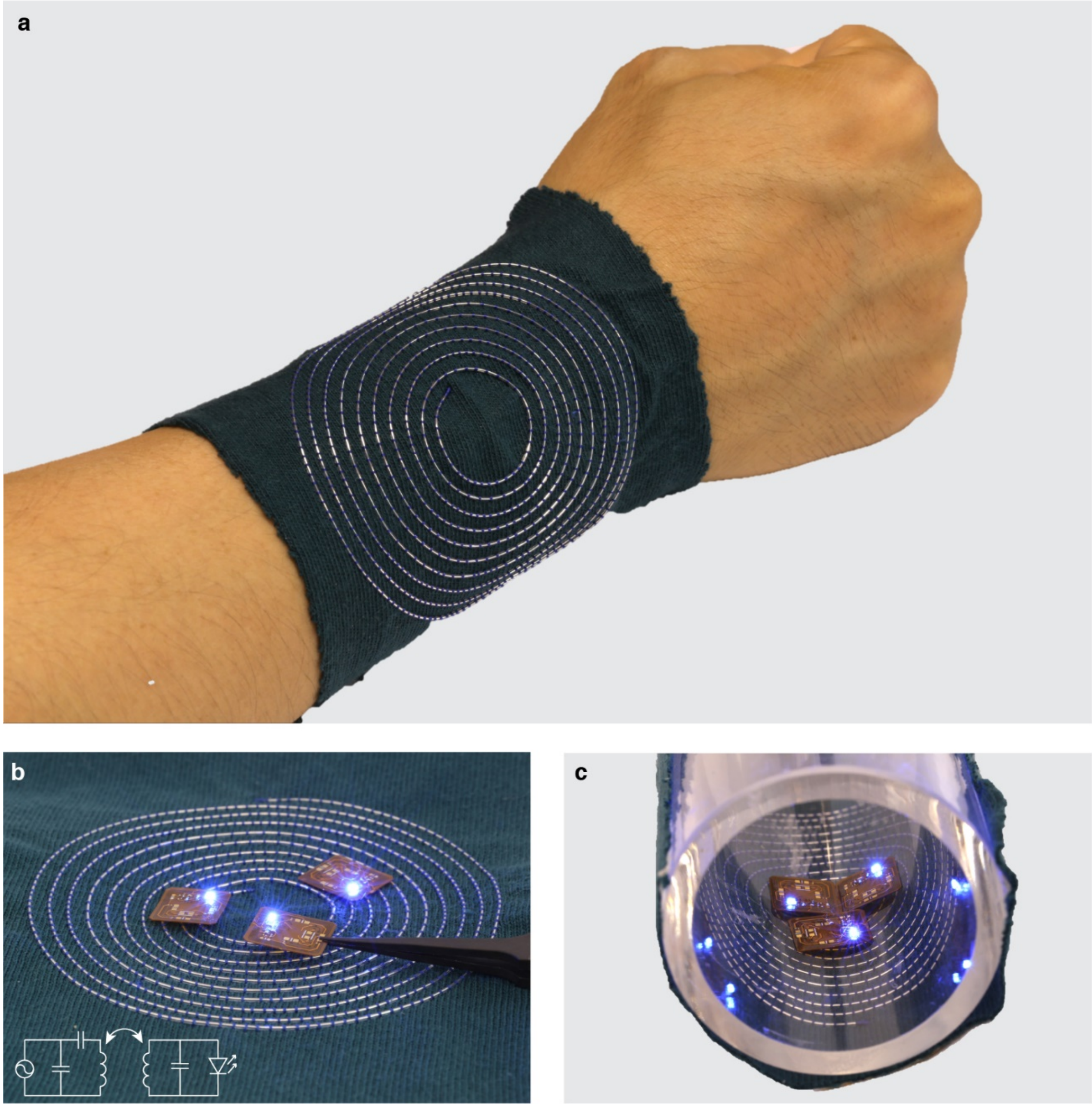

Supplementary Figure 3. Near-field wireless powering functionality. a, An image of a textile transmitter conformally placed on the wrist. **b,c,** The transmitter placed on a flat surface (**b**) and on a cylinder (5-cm diameter) (**c**) provides wireless power to three receivers indicated by blue LEDs. Inset in (**b**) shows the circuit diagram.

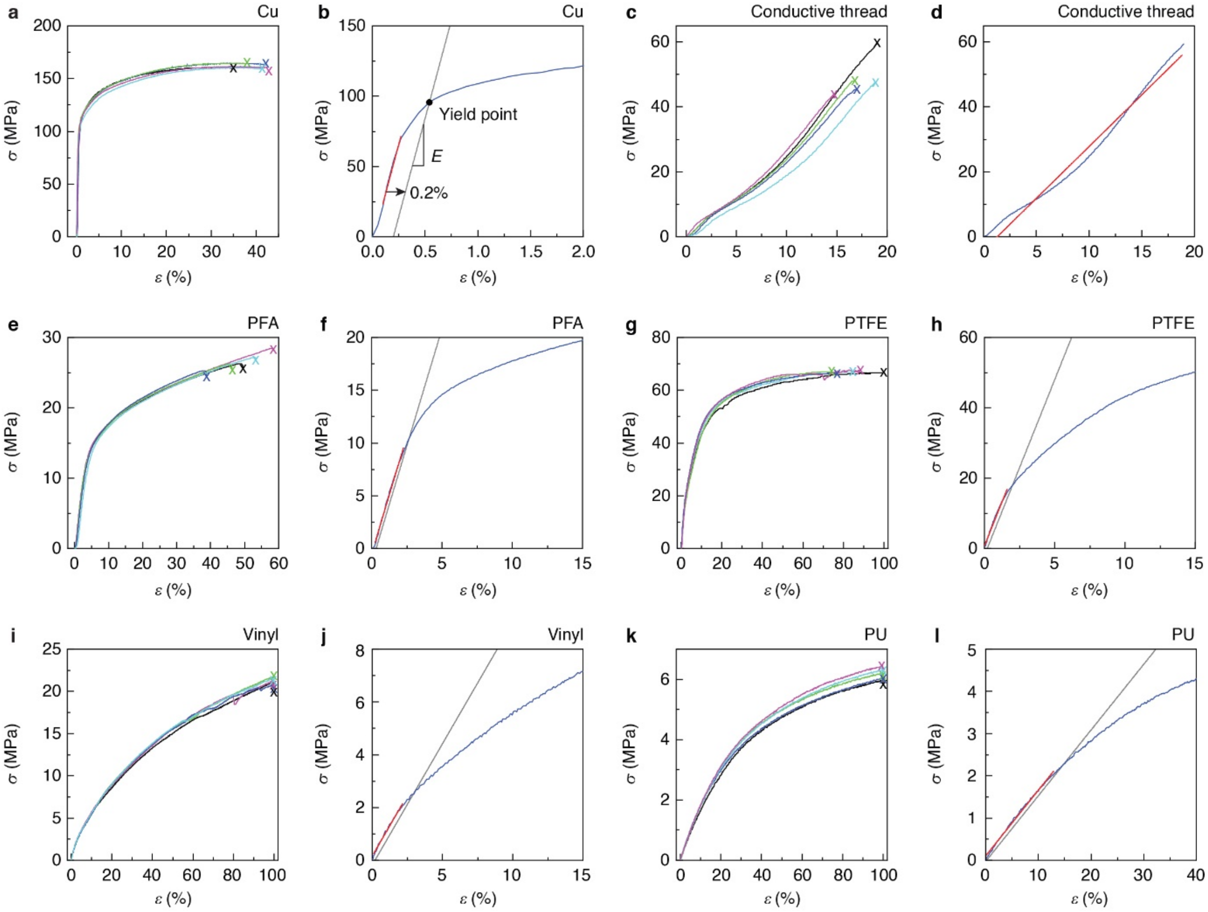

Supplementary Figure 4. Tensile testing. **a**, **c**, **e**, **g**, **i**, **k**, Stress (σ) - strain (ε) curves of Cu wire (**a**), conductive thread (**c**), PFA tubing (**e**), PTFE tubing (**g**), Vinyl tubing (**i**) and PU tubing (**k**). The pulling speed of 1 % s^{-1} (strain per second) is used in all the experiments. **b**, **d**, **f**, **h**, **j**, **l**, Zoom-in of the σ-ε curves. Red line shows the linear fit within the elastic range (Young's modulus E is estimated from the slope). The yield point is the intersection of the green line with the σ-ε curve where the green line is a 0.2 % shift of the red line along the x-axis.

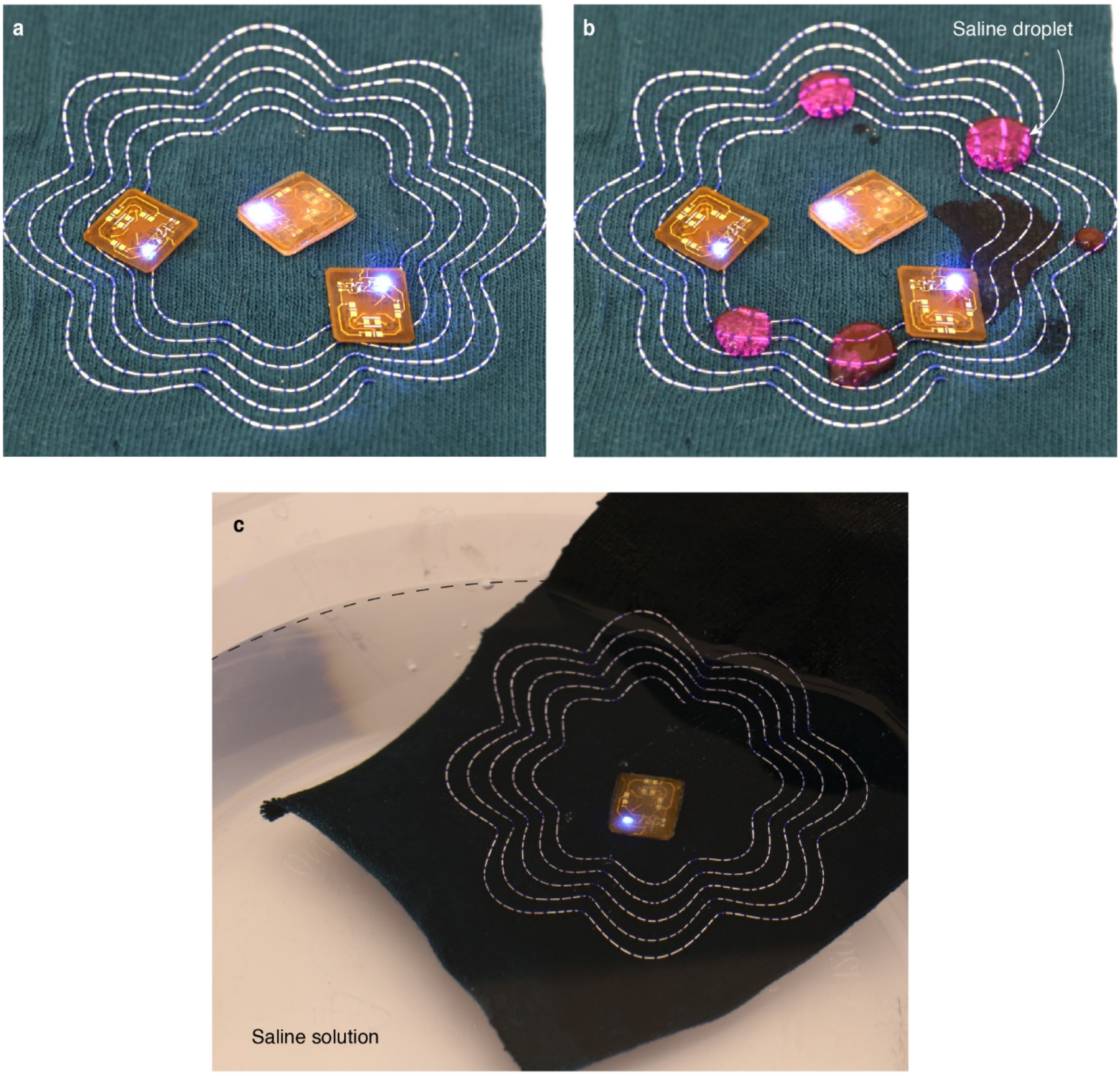

Supplementary Figure 5. Near-field wireless powering in wet environments. a-c, Images of wireless light-emitting devices placed on textile transmitters in a dry environment (**a**), wetted with saline droplets (**b**), and fully immersed in saline solution (**c**). The input power to the transmitter is the same in all images.

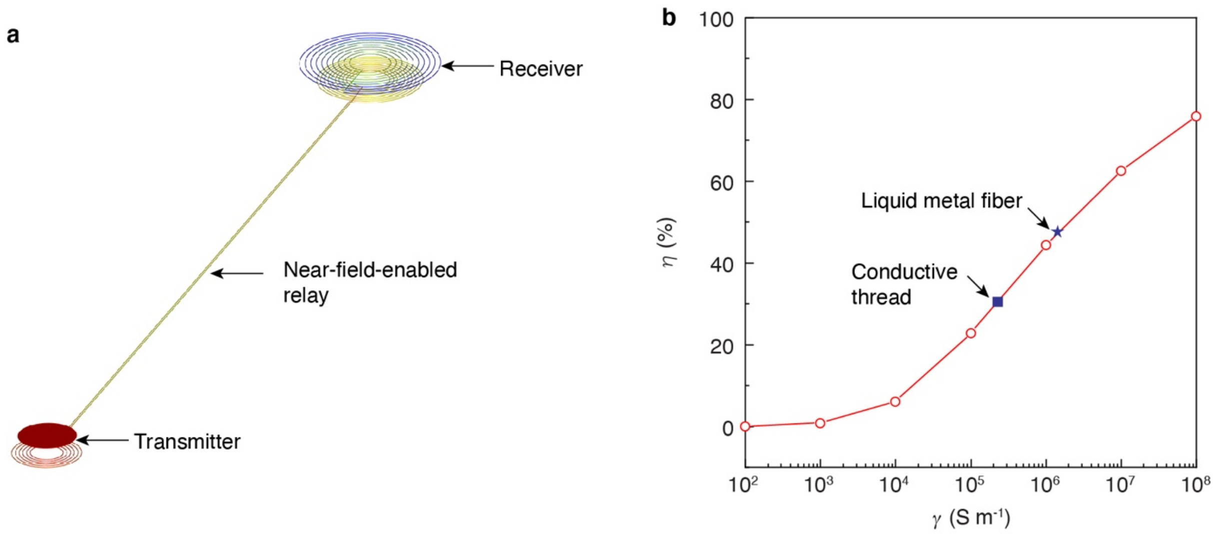

Supplementary Figure 6. Near-field relay performance. a, Near-field relay for inductive wireless power transfer from a transmitter to receiver. The transmitter consists of a 3.1-cm diameter copper coil. The relay is 0.5 m in length (cross section dimensions 0.27 mm×0.27 mm) and has a varying electrical conductivity γ. Receiver coil dimensions: 8-cm in diameter, 3-mm wire gap and 10-turns. Relay coils dimensions: 4-cm diameter, 2-mm wire gap, and 6-turns at the transmitter; 6-cm diameter, 2-mm wire gap and 10-turns at the receiver. The distance of the transmitter and receiver above the relay is 1 cm. **b**, Power transfer efficiency η as a function of the conductivity γ. Black arrows show η corresponding to commercially available conductive threads and liquid metal fibers.

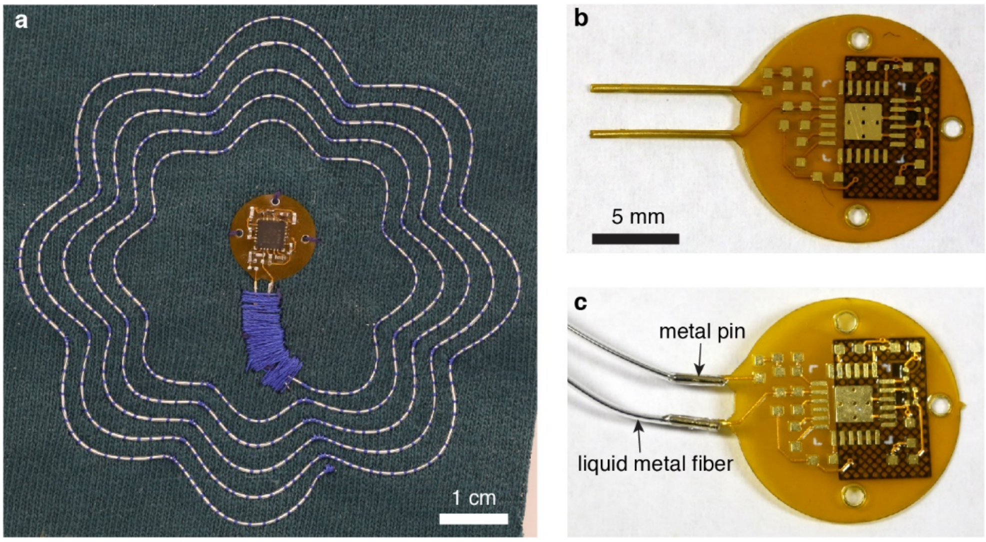

Supplementary Figure 7. Digitally embroidered NFC sensor. a, Image of a clothing-integrated NFC sensor in which digitally embroidered liquid metal fibers form the antenna (8-cm outer diameter). The electrical components are assembled on a polyimide flexible printed circuit board (1.3-cm diameter). **b,c,** Images the flexible printed circuit board. (**b**) The printed circuit board is connected to the antenna by inserting the two tails into the liquid metal fibers and securing the connection with a metal pin (**c**).

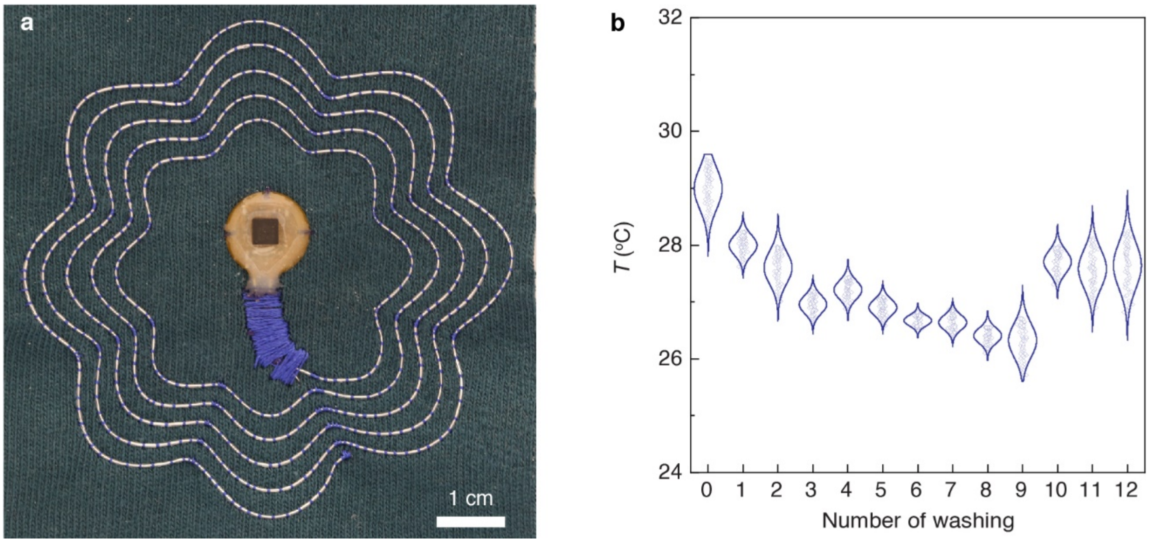

Supplementary Figure 8. Machine washability. a, Image of the clothing-integrated NFC sensor with epoxy encapsulation. The sensor transmits temperature measurements when wirelessly interrogated by an NFC reader. **b**, Environment temperature ($n > 60$ measurements) wirelessly acquired from the sensor as a function of washing cycles. The sensor node is washed together with 2.3 kg clothing using a 50 minutes standard washing program (ISO 6330).

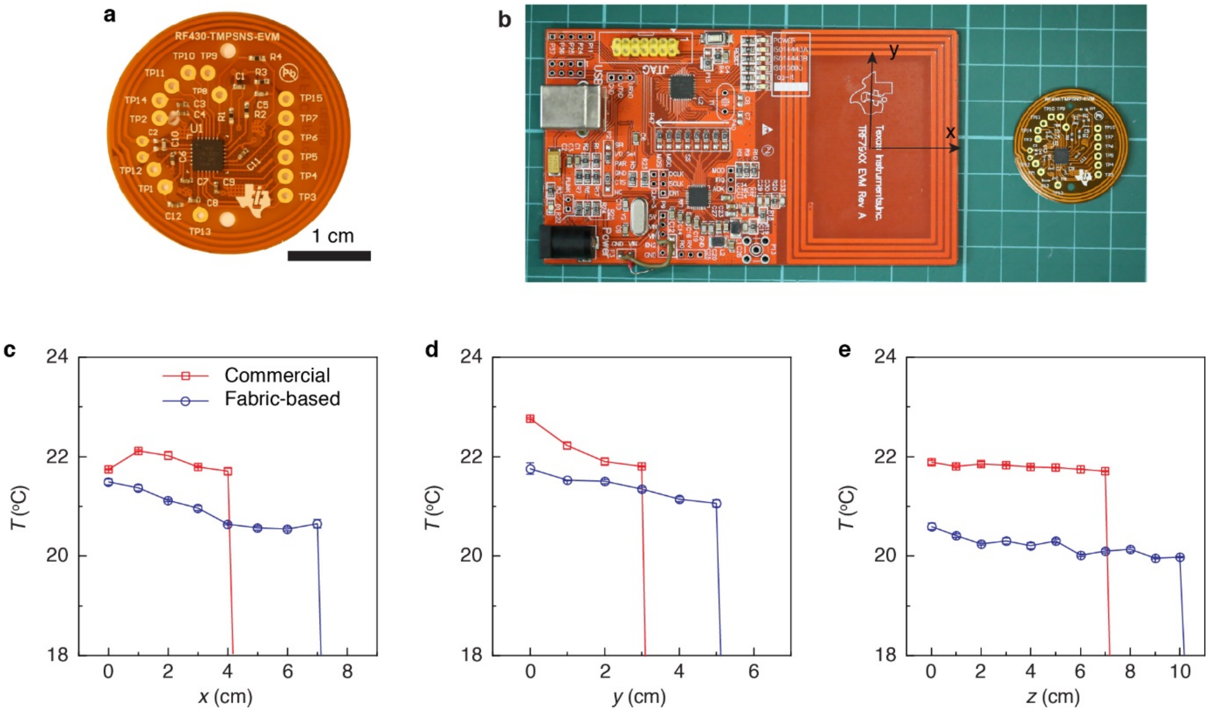

Supplementary Figure 9. Wireless operating range. a, Image of a commercially available NFC sensor (3.1-cm diameter, polyimide substrate, and copper antenna). **b,** Image of the wireless NFC reader and sensor. Coordinate axis shows relative direction of displacement between the sensor and reader. *z* direction is defined as vertical distance. **c-e,** Operating range of the commercially available sensor compared to the clothing-integrated sensor in Supplementary Fig. 7 as a function of displacement in the *x* (**c**), *y* (**d**) and *z* (**e**) directions. The temperature difference is due to different environment conditions during the experiment.

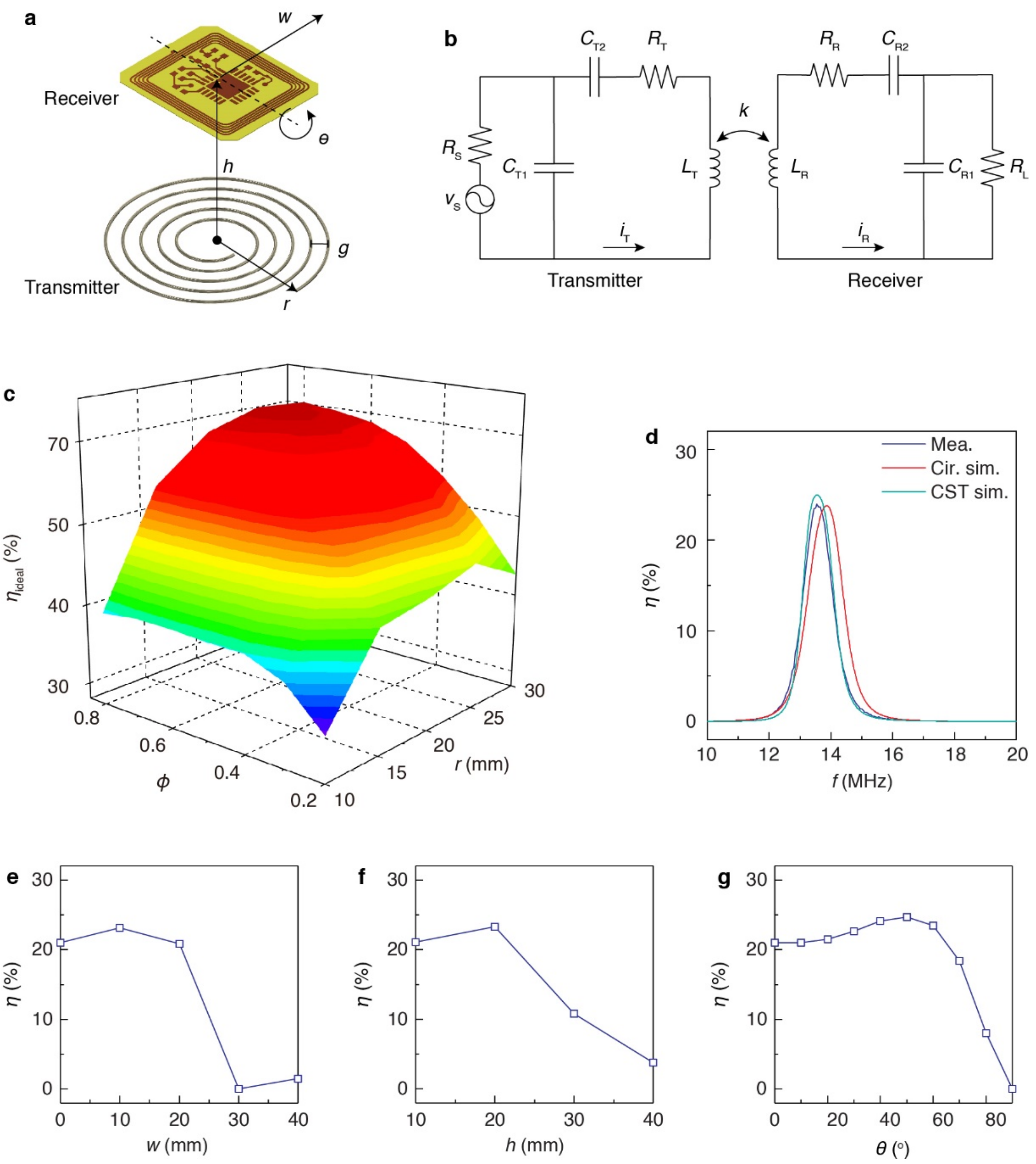

Supplementary Figure 10. Design of the textile transmitter. a, Schematic of the wireless power transfer configuration. The receiver is a rectangular copper coil with dimensions 1.2-cm × 1.2-cm. The parameters are: radius of transmitter *r*, wire gap *g*, number of turns *N*, filled factor ϕ, distance between transmitter and receiver along vertical direction *h* and horizontal direction *w*, rotation angle of the receiver along symmetric axis θ. **b**, Circuit diagram showing tuning capacitors. **c**, Ideal power transfer efficiency η_{ideal} at 13.56-MHz as a function of *r* and ϕ for *g* = 1.5 mm, *h* = 1 cm, *w* = 0 cm, $\theta = 0^o$. The optimized dimensions are *r* = 30 mm, *N* = 13, *g* = 1.5 mm. **d**, Power transfer efficiency η obtained from experiment measurements, circuit simulation and full-wave simulation. **e-f**, η as a function of displacement in the horizontal direction (**e**), vertical direction (**f**) and rotation (**g**).

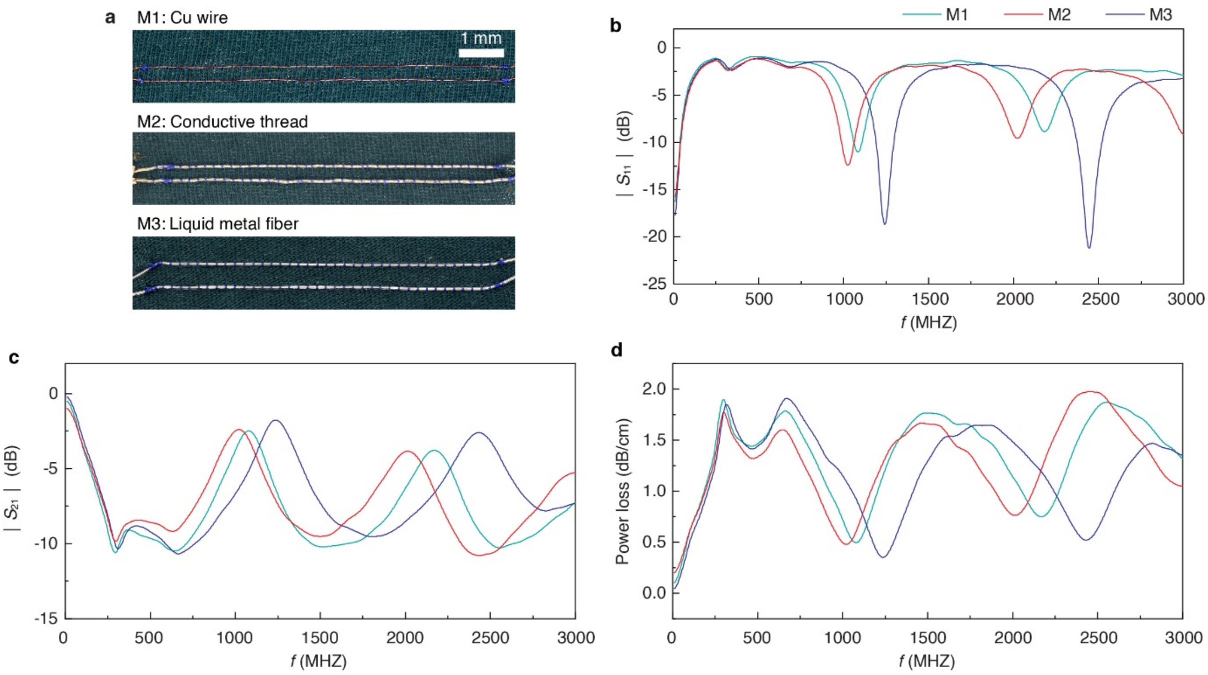

Supplementary Figure 11. Transmitted efficiency across thread-based transmission lines. a, Images of 10-cm length parallel transmission lines integrated with textiles. The thread comprises of copper wire (M1), conductive thread (M2) and liquid metal fiber (M3). **b-c**, Measured scattering parameter $|S_{11}|$ (**b**) and $|S_{21}|$ (**c**) from the embroidered 10-cm transmission lines. **d**, Power loss (insertion loss) per centimeter $(1 - |S_{11}|^2) / (|S_{21}|^2 l)$ through the embroidered transmission lines, where l is length of the transmission line.

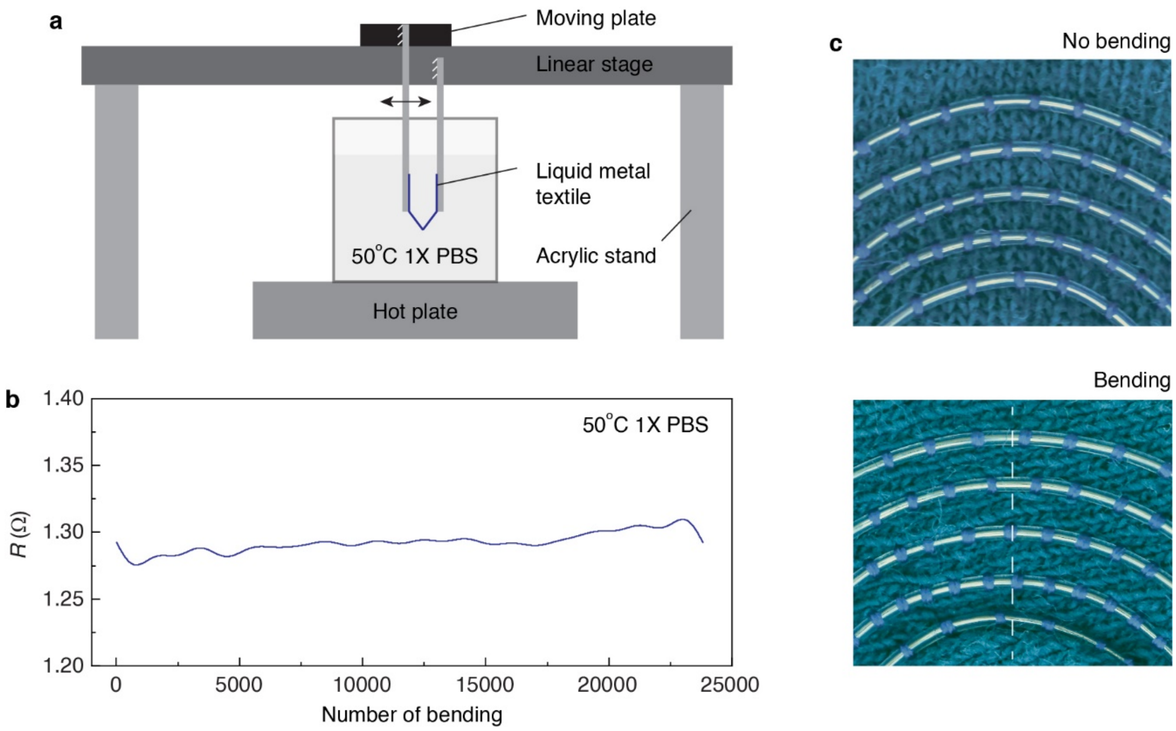

Supplementary Figure 12. Long-term durability of liquid metal textiles bended in sweat analogy. a, Schematic illustration of experimental setups. Liquid metal textile is immersed in 50 °C 1× PBS and cycling bended to a minimum radius of 1 mm. The liquid metal fiber is connected to a digital multimeter to continuously record electrical resistance *R*. **b**, Variation of *R* against the number of bending. The total testing duration is ~13 hours. **c**, Images of liquid metal textile without/with cycling bending. The white dash line indicates the bending position.

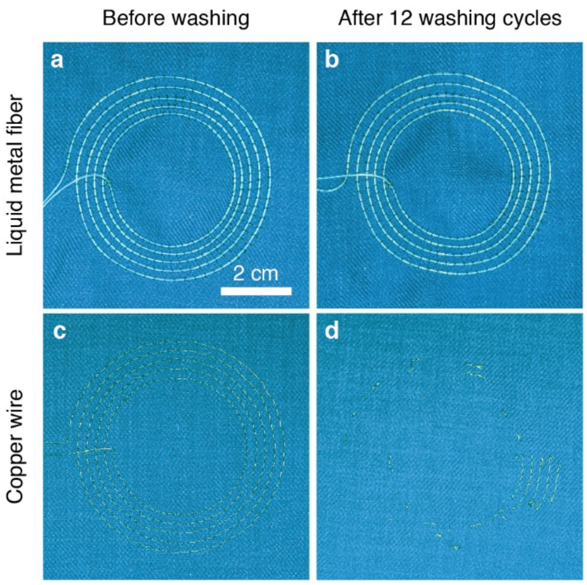

Supplementary Figure 13. Images of textile coils. a-b, Images of liquid metal textile coils before (**a**) and after (**b**) 12 washing cycles. **c-d,** Images of copper textile coils before (**c**) and after (**d**) 12 washing cycles.

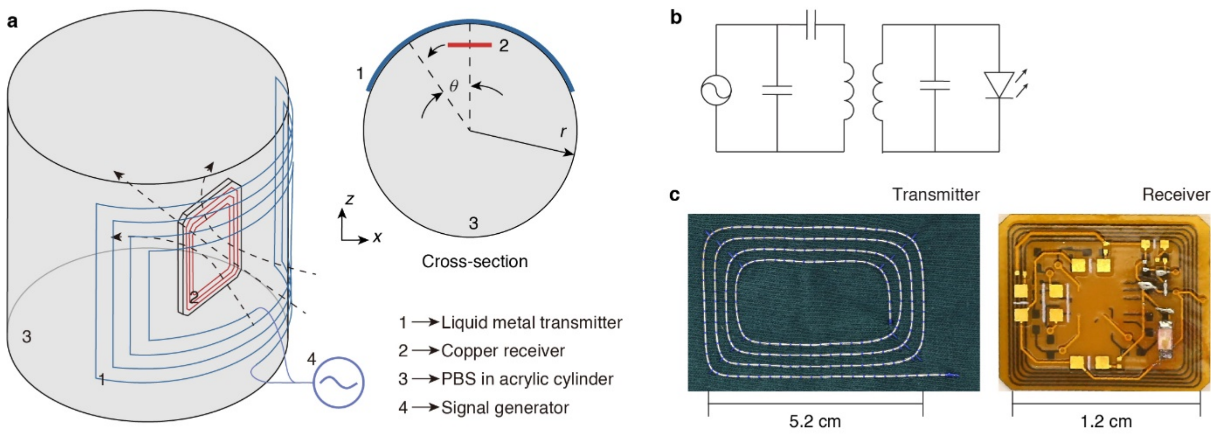

Supplementary Figure 14. Mapping wireless powering area. a, Schematic of the experimental setup. The liquid metal textile transmitter connected with a signal generator is placed conformally on the surface of an acrylic cylinder filled with 1× PBS to mimic biological tissue. The wireless device is simplified by a copper coil connected with a blue LED. The long-exposure images are taken from the top side of the cylinder when the LED is wirelessly activated. **b**, Circuit diagram of the wireless powering system. **c**, Images of the liquid metal textile transmitter (dimensions 5.2-cm × 3.7-cm, 3-mm wire gap, 4 turns) and the wireless device (dimensions 1.2-cm × 1.2-cm, 0.2-mm wire gap, 5 turns).

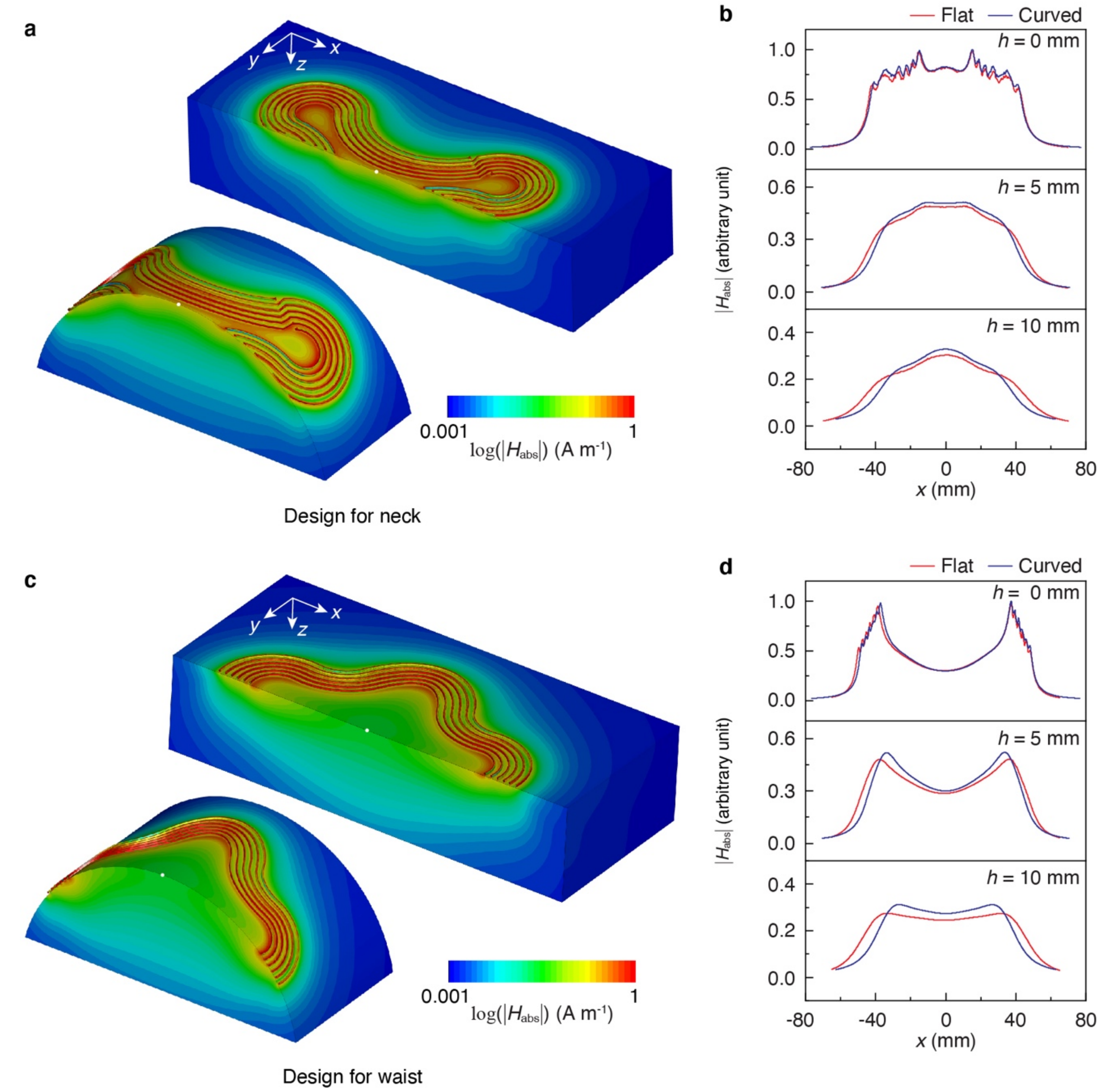

Supplementary Figure 15. Near-field characteristics of the textile transmitter. a, **c**, Simulated magnetic field $|H_{abs}|$ distribution around transmitters designed for the neck (**a**) and back (**c**) placed on a flat and a curved (5-cm radius) body phantom. The transmitter is driven at 13.56 MHz. The cutting plane is set to the center of the skin patch (white dot). **b**, **d,** Field profiles along the cutting plane at varying depth *h* corresponding to the designs in (**a**) and (**c**).

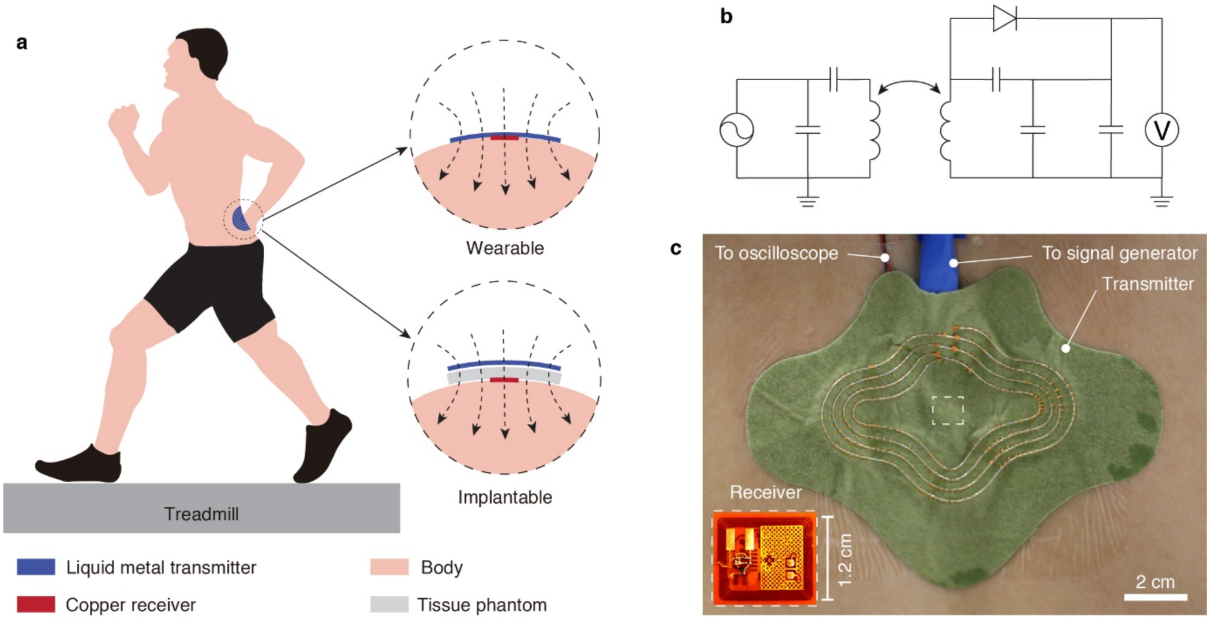

Supplementary Figure 16. On-body wireless powering. a, Schematic of the wearable wireless system. The receiver is placed underneath the textile transmitter with/without a tissue phantom between them, which simulates the implantable and wearable configuration. **b**, Circuit diagram of the wireless powering system. **c**, Images of the textile transmitter attached to the back near the waist area and the receiver. The transmitter is connected to a signal generator. The received voltage is rectified and followed by measuring using an oscilloscope. The dashed rectangle indicates the position of the receiver under the transmitter.

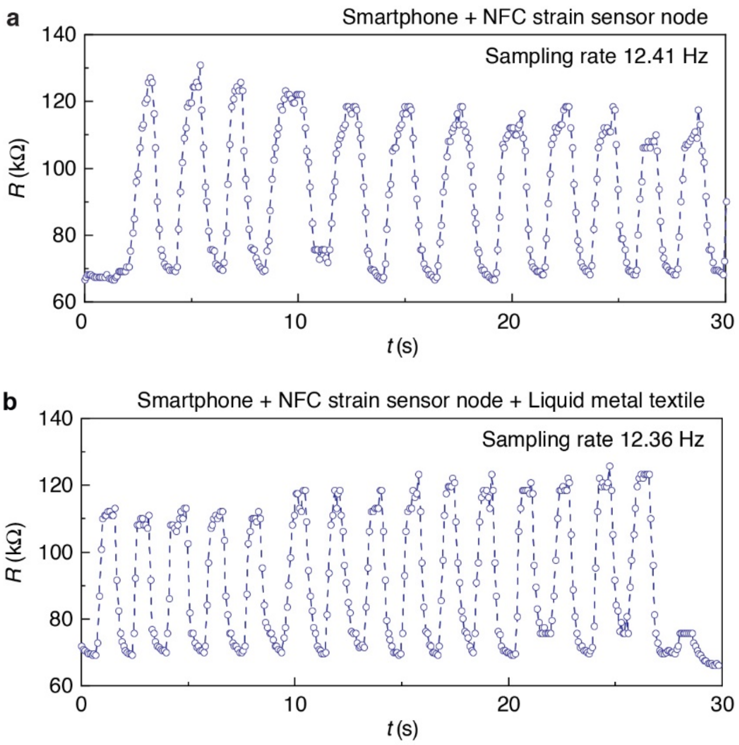

Supplementary Figure. 17. Motion sensing. a, **b**, Wireless systems composed of smartphone and NFC strain sensor node without (**a**) and with (**b**) liquid metal textile as near-field relay are used to detect elbow motion. The NFC strain senor node is mainly composed of an NFC chipset and a custom resistive strain gauge. The sampling rate for both systems is ~12Hz.

References

[S1]. Dickey, M. D. Stretchable and soft electronics using liquid metals. *Advanced Materials* 29, 1606425 (2017).
[S2]. Yan, J., Lu, Y., Chen, G., Yang, M. & Gu, Z. Advances in liquid metals for biomedical applications. *Chemical Society Reviews* 47, 2518–2533 (2018).
[S3]. Gao, J.-Y., Chen, S., Liu, T.-Y., Ye, J. & Liu, J. Additive manufacture of low melting point metal porous materials: Capabilities, potential applications and challenges. *Mater Today* 49, 201–230 (2021).
[S4]. Cole, T., Khoshmanesh, K. & Tang, S.-Y. Liquid metal enabled biodevices. *Advanced Intelligent System* 2000275 (2021).
[S5]. Wang, X. et al. Printed conformable liquid metal e-skin-enabled spatiotemporally controlled bioelectromagnetics for wireless multisite tumor therapy. *Advanced Functional Materials* 29, 1907063 (2019).
[S6]. Kim, J.-H., Kim, S., So, J.-H., Kim, K. & Koo, H.-J. Cytotoxicity of gallium–indium liquid metal in an aqueous environment. *ACS Applied Materials & Interfaces* 10, 17448–17454 (2018).
[S7]. Hallfors, N., Khan, A., Dickey, M. D., & Taylor, A. M. Integration of pre-aligned liquid metal electrodes for neural stimulation within a user-friendly microfluidic platform. *Lab on a Chip* 13(4), 522-526 (2013).
[S8]. Lu, Y. et al. Transformable liquid-metal nanomedicine. *Nature communications* 6(1), 1-10 (2015).
[S9]. Hirsch, A., Dejace, L., Michaud, H. O., & Lacour, S. P. Harnessing the rheological properties of liquid metals to shape soft electronic conductors for wearable applications. *Accounts of chemical research*, 52(3), 534-544 (2019).
[S10] Zhong, J., Kiourti, A., Sebastian, T., Bayram, Y. & Volakis, J. L. Conformal load-bearing spiral antenna on conductive textile threads. *IEEE Antennas and Wireless Propagation Letters* 16, 230–233 (2016).
[S11] Lee, J. H., Dzagbletey, P. A., Jang, M., Chung, J.-Y. & So, J.-H. Flat yarn fabric substrates for screen-printed conductive textiles. *Advanced Engineering Materials* 22, 2000722 (2020).
[S12] Shin, K.-Y., Lee, J. S., Hong, J.-Y. & Jang, J. One-step fabrication of a highly conductive and durable copper paste and its flexible dipole tag-antenna application. *Chemical Communications* 50, 3093–3096 (2014).
[S13] Pan, K. et al. Sustainable production of highly conductive multilayer graphene ink for wireless connectivity and Iot applications. *Nature communications* 9, 1–10 (2018).
[S14] Scid`a, A. et al. Application of graphene-based flexible antennas in consumer electronic devices. *Materials Today* 21, 223–230 (2018).
[S15] Song, R. et al. Flexible graphite films with high conductivity for radio-frequency antennas. *Carbon* 130, 164–169 (2018).
[S16] Han, M. et al. Solution-processed $Ti_3C_2T_x$ Mxene antennas for radio-frequency communication. *Advanced Materials* 33, 2003225 (2021).
[S17] Yang, J. et al. Defect-free, high resolution patterning of liquid metals using reversibly sealed, reusable polydimethylsiloxane microchannels for flexible electronic applications. *Journal of Materials Chemistry C* 5, 6790–6797 (2017).
[S18] Teng, L. et al. Liquid metal-based transient circuits for flexible and recyclable electronics. *Advanced Functional Materials* 29, 1808739 (2019).
[S19]. Dong, C., Leber, A., Gupta, T. D., Chandran, R., Volpi, M., Qu, Y., ... & Sorin, F. High-efficiency super-elastic liquid metal based triboelectric fibers and textiles. *Nature communications*, 11(1), 1-9 (2020).
[S20]. Leber, A., Dong, C., Chandran, R., Gupta, T. D., Bartolomei, N., & Sorin, F. Soft and stretchable liquid metal transmission lines as distributed probes of multimodal deformations. *Nature Electronics*, 3(6), 316-326 (2020).
[S21]. Xiong, Y., Xiao, J., Chen, J., Xu, D., Zhao, S., Chen, S., & Sheng, B. A multifunctional hollow TPU fiber filled with liquid metal exhibiting fast electrothermal deformation and recovery. *Soft Matter*, 17(44), 10016-10024 (2021).
[S22]. Sin, D., Singh, V. K., Bhuyan, P., Wei, Y., Lee, H. M., Kim, B. J., & Park, S. Ultra-stretchable thermo-and mechano-chromic fiber with healable metallic conductivity. *Advanced Electronic Materials*, 2100146 (2021).

[S23]. Xi, W., Yeo, J. C., Yu, L., Zhang, S., & Lim, C. T. Ultrathin and wearable microtubular epidermal sensor for real-time physiological pulse monitoring. *Advanced Materials Technologies*, 2(5), 1700016 (2017).
[S24]. Yu, L., Yeo, J. C., Soon, R. H., Yeo, T., Lee, H. H., & Lim, C. T. Highly stretchable, weavable, and washable piezoresistive microfiber sensors. *ACS applied materials & interfaces*, 10(15), 12773-12780 (2018).
[S25]. Wu, Y. H., Zhen, R. M., Liu, H. Z., Liu, S. Q., Deng, Z. F., Wang, P. P., ... & Liu, L. Liquid metal fiber composed of a tubular channel as a high-performance strain sensor. *Journal of Materials Chemistry C*, 5(47), 12483-12491 (2017).
[S26]. Zhu, S., So, J. H., Mays, R., Desai, S., Barnes, W. R., Pourdeyhimi, B., & Dickey, M. D. Ultrastretchable fibers with metallic conductivity using a liquid metal alloy core. *Advanced Functional Materials*, 23(18), 2308-2314 (2013).
[S27]. Cooper, C. B., Arutselvan, K., Liu, Y., Armstrong, D., Lin, Y., Khan, M. R., ... & Dickey, M. D. Stretchable capacitive sensors of torsion, strain, and touch using double helix liquid metal fibers. *Advanced Functional Materials*, 27(20), 1605630 (2017).
[S28]. Lai, Y. C., Lu, H. W., Wu, H. M., Zhang, D., Yang, J., Ma, J., ... & Dickey, M. D. Elastic multifunctional liquid–metal fibers for harvesting mechanical and electromagnetic energy and as self-powered sensors. *Advanced Energy Materials*, 11(18), 2100411 (2021).
[S29]. Chen, M., Wang, Z., Zhang, Q., Wang, Z., Liu, W., Chen, M., & Wei, L. Self-powered multifunctional sensing based on super-elastic fibers by soluble-core thermal drawing. *Nature communications*, 12(1), 1-10 (2021).
[S30]. Rahimi, R., Yu, W., Ochoa, M., & Ziaie, B. Directly embroidered microtubes for fluid transport in wearable applications. *Lab on a Chip*, 17(9), 1585-1593 (2017).
[S31]. Zheng, L., Zhu, M., Wu, B., Li, Z., Sun, S., & Wu, P. Conductance-stable liquid metal sheath-core microfibers for stretchy smart fabrics and self-powered sensing. *Science Advances*, 7(22), eabg4041 (2021).
[S32]. Yu, Y., Guo, J., Ma, B., Zhang, D., & Zhao, Y. Liquid metal-integrated ultra-elastic conductive microfibers from microfluidics for wearable electronics. *Science Bulletin*, 65(20), 1752-1759 (2020).
