# Supplementary material for: Digitally-embroidered liquid metal electronic textiles for wearable wireless systems
Source: Nat Commun. 2022 Apr 21;13:2190. doi: 10.1038/s41467-022-29859-4 (PMC9023486; doi:10.1038/s41467-022-29859-4)
Supplement: Supplementary file 2 — Description of Additional Supplementary Files [file 41467_2022_29859_MOESM2_ESM.pdf]

Description of Additional Supplementary Files

File Name: Supplementary Movie 1

Description: **Digital embroidery process**. A computer-controlled embroidery machine reproduces a digital pattern created on design software onto a substrate using a liquid metal fiber. For visualization purposes, the substrate shown in the video is a transparent PET film instead of a textile.

File Name: Supplementary Movie 2

Description: **Robustness against mechanical deformation**. An electronic textile created by digital embroidery of liquid metal fibers is subjected to folding and twisting while the electrical resistance is measured using a multimeter. The resistance variation is less than one percent during deformation.

File Name: Supplementary Movie 3

Description: **Washing protocol.** Washing tests follow the ISO 6330 standard for domestic washing and drying. The electronic textile is combined with clothing in a household washing machine to form a 2.3 kg load. An anti-bacterial powder detergent is added and the washing program set to a standard 50-min washing, rinsing, and spinning cycle. Functionality of the electronic textiles after washing is verified by measuring the end-to-end resistance using a multimeter.
